# Deoxycholic acid promotes anxiety- and depression-like behaviors in mice via modulation of the gut microbial metabolite indole-3-propionic acid

**DOI:** 10.3389/fimmu.2026.1840574

**Published:** 2026-06-11

**Authors:** Yulun Wu, Leheng Liu, Dandan You, Tiancheng Mao, Xinbo Zheng, Xin Dai, Xianjun Xu, Xiaowan Wu, Hui Zhou

**Affiliations:** 1 Department of Gastroenterology, Shanghai General Hospital, Shanghai Jiao Tong University School of Medicine, Shanghai, China; 2 Shanghai Key Laboratory of Pancreatic Diseases, Shanghai Jiao Tong University School of Medicine, Shanghai, China; 3 Department of Respiratory Medicine, Center of Infectious Diseases and Pathogen Biology, Key Laboratory of Organ Regeneration and Transplantation of the Ministry of Education, State Key Laboratory for Diagnosis and Treatment of Severe Zoonotic Infectious Diseases, The First Hospital of Jilin University, Changchun, China; 4 Division of Life Sciences and Medicine, Department of Gastroenterology, The First Affiliated Hospital of University of Science and Technology of China (USTC), University of Science and Technology of China, Hefei, China

**Keywords:** anxiety, depression, high fat diet, deoxycholic acid, indole-3-propionic acid, gut microbiota, gut brain axis

## Abstract

**Background:**

High-fat diet (HFD)-associated anxiety- and depression-like behaviors are closely linked to disturbances in the gut–brain axis; however, the peripheral signaling mechanisms and key metabolites involved remain to be elucidated. Deoxycholic acid (DCA), a bile acid elevated by a HFD, has been reported to be associated with abnormal cognitive behaviors in mice. This study aimed to investigate whether HFD-induced anxiety- and depression-like behaviors are regulated by intestinal DCA and its underlying mechanisms.

**Methods:**

Four mouse models were established with different interventions: a low-fat diet (LFD), a HFD, a LFD plus DCA, and a HFD plus the bile acid binder cholestyramine. We performed behavioral phenotyping, brain tissue transcriptome sequencing, fecal 16S rRNA gene sequencing, fecal and serum metabolomics, and intestinal barrier function assessment to clarify the phenotypes and underlying mechanisms. *In vitro* cell experiments, ileal organoid assays, and *in vivo* fecal microbiota transplantation (FMT) were further used for validation.

**Results:**

DCA intervention induced HFD-like anxiety- and depression-like behaviors in the mice, accompanied by reduced levels of the key gut bacterium *Clostridium_sensu_stricto_1* and its metabolite indole-3-propionic acid (IPA) in the gut and serum. IPA supplementation restored circulating IPA levels, upregulated the expression of key genes (*Cyp3a11* and *Abcb1a*) in the cerebral pregnane X receptor (PXR) signaling, ameliorated DCA-induced emotional and behavioral abnormalities, and reversed related gut–brain axis impairments, including downregulated brain barrier-related proteins, morphological changes associated with microglial activation, intestinal barrier damage (reduced goblet cells, downregulated Claudin-1/Occludin), intestinal epithelial oxidative stress and injury, and impaired ileal organoid budding. FMT induced behavioral phenotypes, barrier impairments, reduced serum IPA, and cerebral pathological changes in recipient mice similar to those observed in DCA model mice.

**Conclusions:**

These findings support a potential gut–brain pathway linking HFD-associated luminal DCA elevation to anxiety- and depression-like behaviors in mice. The reduction in IPA levels resulting from the remodeling of gut microbiota triggered by DCA might be the key mediating factor. Targeting abnormal bile acid metabolism or restoring IPA function is a promising intervention strategy for HFD-related emotional and behavioral abnormalities.

## 1 Introduction

Increased consumption of a high-fat diet (HFD) has been associated not only with metabolic disorders but also with neurobehavioral abnormalities. Numerous studies have demonstrated that a HFD can significantly disrupt the gut microbiota and impair internal environmental homeostasis, resulting in intestinal barrier damage and systemic consequences such as metabolic endotoxemia (1, 2). In addition to metabolic phenotypes, accumulating evidence indicates that a HFD is closely associated with the onset and progression of emotional disorders such as anxiety and depression. Studies have shown that continuous HFD consumption in rats induces anxiety- and depression-like behaviors, accompanied by alterations in intracerebral signaling pathways and inflammation-related changes (3). A high-cholesterol diet can also produce similar emotional phenotypes in mice (4), and even prepregnancy/gestational obesity or a HFD in mice may increase the risk of maternal emotional abnormalities (5). However, the key peripheral signals underlying HFD-related emotional disorders remain poorly characterized. In particular, it is still unclear which gut-derived metabolites act as upstream triggers linking HFD exposure to anxiety- and depression-like behaviors caused by gut–brain axis dysfunction.

Research on the gut–brain axis provides a theoretical basis for understanding how diet affects mood. Bidirectional communication between the gut and the brain is achieved through multiple pathways, including neural circuits, microbial metabolites, the immune system, and endocrine signaling (6). Under pathological conditions, gut microbiota dysbiosis can not only alter the production of various microbe-derived metabolites (such as short-chain fatty acids, bile acid derivatives, and tryptophan metabolites) but also can increase intestinal permeability by disrupting the intestinal mucosal barrier. This allows lipopolysaccharide and inflammatory factors to enter the circulation, thus affecting blood–brain barrier (BBB) function, activating microglia, triggering neuroinflammatory responses, and contributing to the development of emotional disorders, including anxiety and depression (7–9). Therefore, identifying key peripheral factors that can drive the continuous cascade of “microbiota–metabolites–barrier–central nervous system (CNS)” changes under HFD conditions is critical for elucidating the mechanisms of anxiety- and depression-like behaviors and developing effective interventions.

Bile acids have been recognized not only as molecules involved in lipid digestion and absorption but also as important signaling molecules involved in the regulation of metabolism and inflammation to contribute to gut–brain communication (10). Primary bile acids can be further transformed into secondary bile acids by specific bacteria in the intestine. Among them, deoxycholic acid (DCA), generated by intestinal bacteria via 7α-dehydroxylation, is a major component of the intestinal bile acid pool (11). Studies have confirmed that DCA considerably regulates ribosomal transcription and amino acid metabolism in the gut microbiota through intracellular accumulation, strongly inhibits the abundance of multiple bacterial species, selectively increases the abundance of certain bacteria, and thus reshapes the composition and metabolic characteristics of the gut microbiota (12). DCA can have a pronounced concentration-dependent effect on the intestine. At high concentrations, the hydrophobicity of DCA can perturb the cell membrane, induce the production of reactive oxygen species (ROS), alter the expression of cell junction-related genes, disrupt tight junctions, and exacerbate barrier dysfunction (13). Our previous studies revealed that a HFD significantly remodeled the bile acid profile and caused an abnormal elevation in the level of fecal DCA, whereas concurrent administration of the bile acid binder cholestyramine (CHO) can reverse HFD-induced DCA enrichment (14, 15). High concentrations of DCA can also promote inflammation-related immune responses and participate in HFD-associated intestinal inflammation (16). Notably, recent evidence also links DCA to cognitive dysfunction in obese mice, supporting the notion that DCA can act beyond the intestine to modulate brain-related phenotypes (17). Although the link between DCA and intestinal injury and inflammation is relatively well established, it remains unclear whether DCA acts as a metabolic component of a HFD or instead as a key upstream peripheral signal contributing to HFD-associated anxiety- and depression-like behaviors.

Among microbiota-derived metabolites, tryptophan metabolites are of particular interest because they represent important interfaces between the gut microbiota and host epithelial, immune, and neural signaling. In particular, microbial indole derivatives have been increasingly recognized to be closely associated with intestinal mucosal homeostasis, anti-inflammatory signaling, barrier regulation, and gut–brain communication (18–20). Among several representative indole metabolites, including indole-3-propionic acid (IPA), indole-3-acetic acid (IAA), and indole-3-lactic acid (ILA), IAA has been reported to participate in intestinal homeostasis and barrier maintenance (21). IPA has been reported to exert antioxidant, anti-inflammatory, and barrier-protective effects through signaling via the pregnane X receptor (PXR)/aryl hydrocarbon receptor pathway, as well as its regulatory effects on the nervous system (22, 23). Emerging clinical and translational evidence suggests that reduced IPA is associated with depressive-like states, although the causality and disease specificity of this association remain incompletely defined (24, 25). Notably, previous studies have demonstrated that HFD-related exposure reduces IPA levels in both feces and plasma (26, 27). However, it remains unclear whether the HFD-associated reduction in IPA synthesis is attributable to gut microbiota remodeling by elevated luminal DCA. Similarly, whether microbiota-derived IPA may represent a downstream effector of DCA that contributes to HFD-induced dysregulation of the gut-brain axis and subsequent neurobehavioral abnormalities also remains to be elucidated.

Thus, we hypothesized that HFD-induced elevation of luminal DCA levels may affect the gut microbiota and alter indole-related tryptophan metabolism to promote gut–brain axis-related barrier and neuroimmune disturbances, contributing to anxiety- and depression-like behaviors.

## 2 Material and methods

### 2.1 Mouse model establishment

Specific pathogen-free grade 8-week-old male C57BL/6J mice (body weight: 18–22 g) were purchased from the Animal Center of Shanghai General Hospital. After 1 week of adaptive feeding, the mice were randomly assigned to different groups, with free access to food and water throughout the experiment. Body weight was measured once a week during the experimental period.

Model 1: Mice were randomly divided into four groups and received continuous dietary intervention for 8 weeks: ① low-fat diet (LFD) group: fed with a 10% fat diet; ② high-fat diet (HFD) group: fed with a 60% fat diet; ③ deoxycholic acid (DCA) group: fed with an LFD mixed with 0.2% (w/w) DCA (D2510, Sigma-Aldrich, USA); ④ cholestyramine (CHO) group: fed with an HFD mixed with 6% (w/w) CHO (C4650, Sigma-Aldrich, USA).

Model 2: Mice were randomly divided into three groups and received continuous intervention for 8 weeks: ① LFD group; ② DCA group; ③ DCA + IPA group: fed with an LFD mixed with 0.2% (w/w) DCA, and IPA. Daily intragastric gavage of 200 μl solution was performed from week 5 to week 8 in mice. The gavage vehicle was normal saline containing 0.5% (w/v) sodium carboxymethyl cellulose (CMC-Na, C835846, Macklin, China). Mice in group ① and ② were gavaged with the vehicle solution, while mice in group ③ received gavage of IPA-containing vehicle solution (IPA, I103959, Aladdin, China) at a dose of 20 mg/kg body weight.

Model 3: Mice were randomly divided into three groups and received continuous intervention for 8 weeks: ① LFD group; ② DCA group; ③ Antibiotic cocktail + fecal microbiota transplantation (FMT) group: fed with an LFD, followed by gut microbiota depletion and FMT intervention. Daily intragastric gavage of 200μL solution was performed from week 5 to week 8 in mice. Mice in group ① and ② were gavaged with normal saline. For mice in group ③, intragastric administration of an antibiotic cocktail was performed during week 5 to deplete the gut microbiota. The antibiotic cocktail consisted of 1g/L ampicillin (A24620, Acmec, China), 1g/L neomycin (N20980, Acmec, China), 1g/L metronidazole (M52060, Acmec, China), and 0.5g/L vancomycin (V76060, Acmec, China). FMT was conducted from week 6 to week 8 with the following procedures: fresh fecal samples were collected daily from mice in group ②, resuspended in normal saline to a final concentration of 0.1 g/mL, and centrifuged at 1500 rpm for 3 min to remove large particulate impurities. The bacterial suspension was adjusted to a final concentration of 1×10⁹ CFU/mL, followed by intragastric administration to the recipient mice.

The intervention doses used above were selected based on our previous work and published mouse studies. Dietary DCA at 0.2% (w/w) was chosen because this concentration has been used in mice to model sustained luminal DCA exposure and to induce gut dysbiosis, fecal bile acid accumulation, and focal ileal/colonic inflammation (28). In our previous studies, the same 0.2% DCA regimen reproducibly increased fecal DCA levels and mimicked key HFD-related intestinal phenotypes, including impaired intestinal stem cell differentiation, reduced goblet/Paneth cell lineages, and mucosal barrier dysfunction (14, 15). CHO was therefore used at 6% (w/w) because, in our previous HFD model, this dose reduced fecal DCA levels and reversed HFD-induced defects in intestinal stem cell differentiation and barrier-related changes (14, 15). IPA was administered by oral gavage at 20 mg/kg body weight because repeated oral supplementation at this dose has shown clear *in vivo* activity in mice, including attenuation of septic injury through enhanced macrophage phagocytosis and improvement of endothelial function and mesenteric vasodilation in HFD-induced obese mice (29, 30). For microbiota depletion before FMT, we used a broad-spectrum antibiotic cocktail consisting of ampicillin (1 g/L), neomycin (1 g/L), metronidazole (1 g/L), and vancomycin (0.5 g/L), which has been widely used in mouse FMT/pseudo-germ-free models (31, 32).

After the completion of behavioral tests for all models, mice were fasted for 12 h and sacrificed by cervical dislocation. Fecal samples, serum, ileal tissues, and brain tissues were collected, and all samples were stored at -80 °C for subsequent detection and analysis.

### 2.2 Behavioral tests

Behavioral tests were performed in the following sequence: Open Field Test (OFT), Elevated Plus Maze Test (EPMT), Tail Suspension Test (TST), Forced Swimming Test (FST), and Sucrose Preference Test (SPT), with a 24-hour interval between two consecutive tests. All behavioral tests were conducted and analyzed under blinded conditions. Group information was concealed from the experimenters during behavioral testing, and video files were coded before trajectory annotation and behavioral quantification.

OFT: Each mouse was placed in the central area of an open field arena with dimensions of 40 × 40 × 40 cm, and its movement trajectory was recorded for 10 min using a camera. The primary analyzed indicators included the number of entries into the central area and the total duration of stay in the central area. Total distance traveled was additionally quantified as a secondary comparative parameter to evaluate general locomotor activity.

EPMT: The test was conducted using a cross-shaped maze elevated 40 cm above the ground, consisting of two open arms (30 × 5 cm), two closed arms (30 × 5 cm with 15 cm-high surrounding walls), and a central platform (5 × 5 cm). Each mouse was placed in the central platform with its head facing to an open arm, and its movement trajectory was recorded for 6 min. The number of entries into the open arms and the total duration of stay in the open arms were analyzed as primary indicators.

Trajectory data from OFT and EPMT were annotated using the CVAT labeling tool, and the above behavioral indicators were quantified using Python software (version 3.14).

TST: The tail of each mouse was fixed with medical adhesive tape at a position 1 cm from the tail tip, and a rigid plastic tube was sleeved over the tail root to prevent climbing behavior during the test. Mice were suspended vertically, and video recording was performed for 6 min. The total immobility time during the last 4 min of the test was counted and statistically analyzed.

FST: Each mouse was individually placed into a transparent beaker filled with 800 mL of clean water maintained at 23–25 °C, and video recording was conducted for 6 min. The total immobility time during the last 4 min of the test was quantified. If most or individual mice failed to maintain a floating state during the experiment, the batch of tests was terminated immediately.

SPT: A two-bottle choice paradigm was adopted for this test. One bottle contained 2% (w/v) sucrose solution, and the other contained pure water, with an initial volume of approximately 40 mL for each bottle. A 24-hour adaptive training was performed prior to the formal test, and the positions of the two bottles were swapped at the 12th hour of training. At the onset of the formal test, the bottle positions were swapped again, the liquid in each bottle was replenished to approximately 40 mL, and the exact initial volume was recorded. The residual volume of liquid in each bottle was measured after 24 hours of the formal test. The sucrose preference rate was calculated using the following formula: Sucrose preference rate = sucrose solution intake/total liquid intake.

### 2.3 Fecal 16S rRNA gene sequencing and bioinformatics analysis

Fecal samples were collected and stored at -80 °C prior to analysis. Microbial genomic DNA was extracted using the HiPure Stool DNA Kit (Magen, Guangzhou, China) according to the manufacturer’s instructions. DNA quality and concentration were assessed using a NanoDrop 2000 spectrophotometer and agarose gel electrophoresis.

The V3–V4 hypervariable regions of the bacterial 16S rRNA gene were amplified using barcoded universal primers 341F (5′-CCTACGGGNGGCWGCAG-3′) and 806R (5′-GGACTACHVGGGTATCTAAT-3′). PCR amplification was performed in a 50 μl reaction system using Q5 High-Fidelity DNA Polymerase (New England Biolabs, USA) under the following conditions: initial denaturation at 95 °C for 5 min; 30 cycles of 95 °C for 1 min, 60 °C for 1 min, and 72 °C for 1 min; followed by a final extension at 72 °C for 7 min. PCR products were purified using AMPure XP Beads (Beckman Coulter, USA) and quantified with a Qubit 3.0 Fluorometer (Thermo Fisher Scientific, USA).

Sequencing libraries were constructed using the Illumina DNA Prep Kit (Illumina, USA) and validated by quantitative PCR on an ABI StepOnePlus Real-Time PCR System (Applied Biosystems, USA). Qualified libraries were pooled and sequenced on the Illumina NovaSeq 6000 platform using the paired-end 250 bp (PE250) mode.

Raw reads were quality-filtered using fastp, and paired-end reads were merged using FLASH. High-quality sequences were processed using the DADA2 pipeline within QIIME2, including dereplication, error correction, denoising, and chimera removal to generate amplicon sequence variants (ASVs). Taxonomic assignment was performed against the SILVA database (version 138.1) using a naïve Bayesian classifier with a confidence threshold of 0.8.

### 2.4 Targeted tryptophan metabolomics analysis

Fecal samples were stored at -80 °C until analysis. Approximately 50 mg of each sample was weighed and extracted with 500 μl methanol containing 20 μl internal standard solution (250 ng/mL). Samples were vortexed for 3 min and incubated at −20 °C for 30 min to facilitate protein precipitation. After centrifugation at 12,000 rpm for 10 min at 4 °C, 250 μl of the supernatant was transferred to a new tube and centrifuged again. Finally, 150 μl of the supernatant was transferred to LC vials for analysis.

Targeted metabolite quantification was performed using an ultra-performance liquid chromatography–tandem mass spectrometry (UPLC–MS/MS) system consisting of an ExionLC™ AD UPLC coupled to a QTRAP^®^ 6500+ mass spectrometer (SCIEX). Chromatographic separation was achieved using a Waters ACQUITY UPLC HSS T3 C18 column (1.8 μm, 100 mm × 2.1 mm). The mobile phases consisted of solvent A (water with 0.1% formic acid) and solvent B (acetonitrile with 0.1% formic acid). The flow rate was set at 0.35 mL/min, column temperature at 40 °C, and injection volume at 2 μl. The gradient elution program was as follows: 0–1 min, 90% A; 1–6 min, linear gradient to 5% A; 6–7 min, 5% A; 7.1–10 min, re-equilibrated to 90% A.

Mass spectrometric detection was performed in multiple reaction monitoring (MRM) mode. Metabolites were identified based on retention time and characteristic ion transitions, and quantified using external standard calibration curves. Data preprocessing, normalization, and statistical analyses were performed prior to downstream multivariate and enrichment analyses.

### 2.5 Untargeted metabolomics analysis

Serum samples were stored at -80 °C until analysis. Serum metabolites were extracted using cold organic solvent precipitation (methanol/acetonitrile), followed by vortexing and centrifugation. The supernatant was collected, dried under vacuum, and reconstituted prior to LC-MS/MS analysis.

Metabolomic profiling was performed using an ultra-high performance liquid chromatography system (Vanquish, Thermo Scientific, USA) coupled to a high-resolution mass spectrometer (Q Exactive HF-X, Thermo Scientific, USA). Data were acquired in both positive and negative electrospray ionization modes (ESI+ and ESI−). The main MS parameters were as follows: spray voltage 3500 V (ESI+) and 3200 V (ESI−); sheath gas 30 Arb; auxiliary gas 5 Arb; ion transfer tube temperature 320 °C; vaporizer temperature 300 °C. MS1 spectra were acquired over a mass range of 75–1000 m/z at a resolution of 35,000 with an AGC target of 1.0E+06. MS2 spectra were collected at a resolution of 17,500 with an AGC target of 2.0E+05. Data-dependent acquisition was performed with Top 10 precursor ions per cycle and collision energies of 30, 40, and 50 eV. Dynamic exclusion was set to 3 s.

Raw mass spectrometry data were converted to mzML format using ProteoWizard and processed in XCMS for peak detection, retention time alignment, and correction. Features with missing values >50% were removed. Remaining missing values were imputed using K-nearest neighbor (KNN) or replaced with 1/5 of the minimum detected value when appropriate. Signal drift correction was performed using support vector regression (SVR). Metabolite annotation was conducted by matching against an in-house database combined with public metabolite databases. Only metabolites with an identification score ≥0.5 and coefficient of variation (CV) <30% in quality control samples were retained for downstream analysis. Positive and negative ion mode datasets were merged for subsequent statistical and pathway analyses.

### 2.6 Measurement of IPA by ELISA

Serum IPA concentrations were measured using a competitive ELISA kit (BE00310D1, BIOAGRIO, China) according to the manufacturer’s protocol. All samples were analyzed in duplicate.

Briefly, 40 μl of sample diluent and 10 μl of sample (final 5-fold dilution) were added to each antibody-coated well. For standard wells, 50 μl of standard solution was added. Subsequently, 50 μl of HRP-conjugated IPA reagent was added to each well except the blank control. Plates were sealed and incubated at 37 °C for 60 min. After incubation, plates were washed five times with diluted wash buffer (each wash with 30 s soaking). Then, 50 μl of substrate solution A and 50 μl of substrate solution B were added to each well and incubated at 37 °C in the dark for 15 min. The reaction was terminated by adding 50 μl stop solution to each well, resulting in a color change from blue to yellow.

Absorbance was measured at 450 nm using a microplate reader within 15 min after adding the stop solution. A standard curve was generated from the provided standards, and sample concentrations were calculated by regression analysis and corrected by the dilution factor.

### 2.7 RNA-seq analysis

Total RNA was extracted from mouse whole brain tissues and HT-29 cells using TRIzol^®^ Reagent according to the manufacturer’s protocol. RNA concentration and purity were measured with a NanoDrop 2000 spectrophotometer (Thermo Fisher Scientific, USA), and RNA integrity was assessed using an Agilent 5300 Bioanalyzer (Agilent Technologies, USA). Only high-quality RNA samples (RQN > 4.5, concentration ≥ 20 ng/μl) were used for subsequent library construction.

RNA-seq libraries were prepared using the Illumina^®^ Stranded mRNA Prep Kit (Illumina, San Diego, CA, USA). Briefly, mRNA was enriched from total RNA using oligo(dT) magnetic beads and fragmented into approximately 300 bp fragments. First-strand cDNA was synthesized using random hexamer primers, followed by second-strand cDNA synthesis to generate double-stranded cDNA. The cDNA fragments were subjected to end repair, 3′ A-tailing, and adapter ligation. After purification and size selection (300–400 bp) using magnetic beads, libraries were amplified by PCR to obtain the final sequencing libraries. Library concentration was quantified using Qubit 4.0 fluorometry, and library quality was verified prior to sequencing.

Paired-end sequencing (150 bp) was performed on the Illumina NovaSeq X Plus platform according to the manufacturer’s instructions. Raw reads were processed to remove adapter sequences and low-quality reads using fastp, and the resulting clean reads were aligned to the reference genome (Mus musculus for mouse brain samples and Homo sapiens for HT-29 cells) using HISAT2 with default parameters. Gene expression levels were subsequently quantified for downstream analysis.

### 2.8 Protein extraction and western blot

Total protein was isolated from tissue samples using RIPA buffer (PC101, EpiZyme, China) supplemented with protease and phosphatase inhibitor cocktail (P1050, Beyotime, China). Samples were homogenized using a high-throughput tissue grinder at 70Hz for 60s, and the homogenization step was performed twice to ensure complete disruption. The lysates were kept on ice for 1h to facilitate protein extraction, followed by centrifugation at 12,000 rpm for 15min at 4 °C. The clarified supernatants were collected, and protein concentrations were determined using a BCA assay kit (20201ES76, Yeasen, China).

Protein samples were combined with 5× SDS loading buffer (LT101, EpiZyme, China) at a ratio of 4:1 and heated at 100 °C for 10 min to achieve denaturation. After cooling, samples were stored at -80 °C until analysis. Equal quantities of protein were separated by electrophoresis on 10% SDS–polyacrylamide gels and subsequently transferred onto PVDF membranes (ISEQ00010, Millipore, USA). Membranes were blocked with 5% nonfat milk for 1h at room temperature and then incubated overnight at 4 °C with primary antibodies against Claudin-1 (1:1000, A21971, ABclonal, China), Occludin (1:5000, 27260-1-AP, Proteintech, China), and β-actin (1:1000, GB15003, Servicebio, China). After washing with TBST, membranes were incubated with Peroxidase-conjugated goat anti-rabbit IgG secondary antibody (1:20000, 111-035-003, Jackson ImmunoResearch, USA) for 1h at room temperature. Protein bands were detected using an enhanced chemiluminescence (ECL) reagent, and band intensities were quantified with ImageJ software (version 1.54g).

### 2.9 Immunofluorescence

Total mouse brain tissues were harvested and immediately fixed in 4% (w/v) paraformaldehyde for 24 h at room temperature, followed by gradient ethanol dehydration, xylene clearance, and paraffin embedding. Tissues were serially sectioned at a thickness of 20 μm, then deparaffinized in xylene and rehydrated through a graded ethanol series. For antigen retrieval, sections were immersed in citrate buffer (pH 6.0), heated in a microwave for 15 min, and cooled naturally to room temperature. After rinsing with phosphate-buffered saline (PBS), sections were blocked with 5% bovine serum albumin (BSA) for 1 h at room temperature to eliminate non-specific binding. Sections were then incubated overnight at 4 °C with the primary antibody against IBA1 (1:200, A19776, ABclonal, China). After three washes with PBS, sections were incubated with Alexa Fluor 488-conjugated goat anti-rabbit secondary antibody (1:200, 34206ES60m, Yeasen, China) for 1 h at room temperature in the dark. Nuclei were counterstained with 4’,6-diamidino-2-phenylindole (DAPI, 1:1000, 40728ES03, Yeasen, China) for 3 min, followed by dehydration, clearance, and mounting with anti-fluorescence quenching medium.

Z-stack fluorescent images were acquired using a Zeiss LSM980 laser scanning confocal microscope (Carl Zeiss, Germany). For quantitative analysis, 5 random non-overlapping visual fields were selected for each sample in a blinded manner, and the fluorescence intensity of IBA1-positive cells was analyzed using ImageJ software (version 1.54g).

### 2.10 Periodic Acid-Schiff staining

Fresh mouse terminal ileal tissues were harvested and immediately fixed in 4% (w/v) paraformaldehyde for 24 h at room temperature. After gradient ethanol dehydration, xylene clearance and paraffin embedding, the tissues were serially sectioned at a thickness of 4 μm. For Periodic Acid-Schiff (PAS) staining, sections were deparaffinized, rehydrated, and incubated with 0.5% periodic acid solution for 15 min in the dark, followed by rinsing and 20 min of Schiff reagent staining at room temperature. Sections were then counterstained with hematoxylin, dehydrated in gradient ethanol, and mounted with neutral resin for observation under an optical microscope.

Blinded quantitative analysis of ileal goblet cells was performed: 5 random non-overlapping visual fields (200× magnification) were selected for each sample, and the number of positive goblet cells in each villus-crypt unit was counted. The average number of goblet cells per villus-crypt unit was calculated for subsequent statistical comparison between groups.

### 2.11 HT-29 cell culture and sequential drug intervention

Human colon adenocarcinoma HT-29 cell line (CL0118, Pricella, China) was routinely cultured in high-glucose Dulbecco’s modified Eagle medium (DMEM) supplemented with 10% fetal bovine serum and 1% penicillin-streptomycin, and maintained in a humidified incubator at 37 °C with 5% CO_2_. Cells in the logarithmic growth phase were seeded into culture plates, and intervention experiments were performed after complete cell adherence.

Cells were randomly divided into three groups with a uniform total incubation duration of 36 h to ensure consistent experimental conditions: ① Normal Control (NC) group: treated with equal volume of DMSO vehicle throughout the incubation period; ② DCA group: treated with DCA at a final concentration of 0.1 mM for the full 36 h; ③ DCA + IPA group: pre-incubated with 0.1 mM DCA for 12 h first, then IPA was added to the medium at a final concentration of 0.1 mM, followed by co-incubation of DCA and IPA for another 24 h. The final concentration of DMSO was consistent across all groups to eliminate solvent-related interference. After the intervention, cells were harvested for subsequent analysis.

The intervention conditions were selected based on published intestinal epithelial cell studies and our previous experiments. DCA at 0.1 mM was chosen because micromolar DCA exposure has been used in HT-29 models, and our previous HT-29 study also used 100 μM DCA (15). IPA at 0.1 mM was chosen because 100 μM lies within the concentration range tested in intestinal epithelial cells (33). The sequential design of 12 h DCA pre-incubation followed by 24 h co-incubation with DCA and IPA was determined by preliminary optimization experiments.

### 2.12 ROS detection

Intracellular ROS levels in adherent cells were measured using a Reactive Oxygen Species Assay Kit (G1706, Servicebio, China) based on the DCFH-DA fluorescent probe. Cells were seeded in culture plates one day prior to the experiment to achieve 50–70% confluence at the time of detection. After the designated treatments, culture medium was removed and cells were washed twice with phosphate-buffered saline (PBS) to eliminate serum interference. DCFH-DA was diluted 1:1000 in serum-free medium to prepare the working solution. Cells were incubated with the DCFH-DA working solution at 37 °C for 30 min in the dark. Following incubation, cells were washed three times with PBS to remove excess probe and maintained in PBS for fluorescence detection. Fluorescence signals were detected immediately using a fluorescence microscope or flow cytometer with an excitation wavelength of 488 nm and an emission wavelength of 525 nm (FITC channel settings). The mean fluorescence intensity was quantified to evaluate intracellular ROS levels. All experiments were performed in at least three independent replicates.

### 2.13 RNA extraction and quantitative real-time PCR

Total RNA was extracted from the intervened HT-29 cells and mouse brain tissues using TRIzol reagent (9109, Takara, Japan) following the manufacturer’s standard protocol. Briefly, the culture medium was discarded, and cells were rinsed twice with PBS, followed by the addition of TRIzol reagent for complete cell lysis at room temperature. Chloroform was added to the cell lysate, and the mixture was centrifuged to separate the aqueous phase containing total RNA. RNA was precipitated with isopropanol, washed twice with 75% ice-cold ethanol, air-dried at room temperature, and finally dissolved in RNase-free water. The concentration and purity of total RNA were determined using a NanoDrop spectrophotometer, and only RNA samples with an A260/A280 ratio of 1.8–2.0 were used for subsequent reverse transcription.

A total of 2000 ng of qualified total RNA was reverse-transcribed into complementary DNA (cDNA) using the HyperScript III RT SuperMix Kit (R202-02, EnzyArtisan, China) according to the kit instructions. Quantitative real-time PCR was subsequently performed in a 10 μl reaction system using the Universal SYBR qPCR Mix Kit (Q204-05, EnzyArtisan, China). In HT-29 cells, the mRNA expression levels of *GCLC*, *CYP3A4*, and *ABCB1* were measured using human *GAPDH* as the internal reference. The primer sequences were as follows: *GCLC* forward, GGAGGAAACCAAGCGCCAT; reverse, CTTGACGGCGTGGTAGATGT; *CYP3A4* forward, TGTTTTCAGCCCATCTCCTTTC; reverse, GCATCGAGACAGTTGGGTGTTG; *ABCB1* forward, TTTCCACTAAAGTCGGAGTATCTTC; reverse, GTCCCCTTCAAGATCCATTCC; *GAPDH* forward, TGTGGGCATCAATGGATTTGG; reverse, ACACCATGTATTCCGGGTCAAT. In mouse brain tissues, the mRNA expression levels of *Drd2*, *Fos, Cyp3a11, and Abcb1a* were measured using mouse *Gapdh* as the internal reference. The primer sequences were as follows: *Drd2* forward, ACCTGTCCTGGTACGATGATG; reverse, GCATGGCATAGTAGTTGTAGTGG; *Fos* forward, AACAGATCCGAGCAGCTTCTA; reverse, TTTTGAGCTTCAACCGGCATC; *Cyp3a11* forward, GTCAAACGCCTCTCCTTGCTG; reverse, CGCCGGTTTGTGAAGACAGAA; *Abcb1a* forward, TCCTCACCAAGCGACTCCGATA; reverse, ACTTGAGCAGCATCGTTGGCGA; *Gapdh* forward, AATGGATTTGGACGCATTGGT; reverse, TTTGCACTGGTACGTGTTGAT. Relative mRNA expression levels were calculated using the 2^−ΔΔ^Ct method.

### 2.14 Lactate dehydrogenase release detection

Lactate dehydrogenase (LDH) release was measured using an LDH Release Assay Kit (WST-8 method, C0019, Beyotime, China) according to the manufacturer’s instructions. Briefly, adherent cells were seeded in 96-well plates and treated as indicated. After treatment, culture supernatants were collected and centrifuged at 400×g for 5 min to remove debris.

For detection, 1 μl of supernatant was added to each well and adjusted to 100 μl with LDH Release Assay Buffer. Subsequently, 100 μl of freshly prepared LDH working solution (LDH Release Assay Buffer: Chromogen Solution = 91: 9, v/v) was added and incubated at 37 °C for 30 min in the dark. The reaction was terminated by adding 20 μl Stop Solution. Absorbance was measured at 450 nm using a microplate reader. LDH activity was calculated based on a standard curve and normalized to the dilution factor. Background signals from medium-only control wells were subtracted.

### 2.15 Mouse ileal organoid culture

Fresh terminal ileal tissues were harvested from C57BL/6J mice, rinsed with pre-chilled PBS to remove intestinal contents, longitudinally incised and cut into 0.5 cm segments. The tissue segments were incubated with 5 mM ethylenediaminetetraacetic acid (EDTA) in pre-chilled PBS at 4 °C for 1 h with gentle shaking to release intestinal crypts. The tissue suspension was filtered through a 70 μm cell strainer to remove undigested debris, followed by centrifugation at 1200 rpm for 5 min at 4 °C to collect the crypt precipitate.

Isolated crypts were resuspended in growth factor-reduced Matrigel (356234, Corning, USA), and 20 μL of the Matrigel-crypt mixture was seeded into each well of a 48-well plate. After Matrigel solidification at 37 °C for 15 min, 200 μL of Organoid Growth Medium (WM-M-01, OuMel, China) was added to each well. Organoids were cultured in a humidified 37 °C incubator with 5% CO_2_, with the culture medium refreshed every 48 h. Well-developed organoids with intact epithelial structure and stable budding were selected for subsequent drug intervention.

Organoids were randomly divided into 3 groups with a total intervention duration of 96 h: ① NC group: treated with equal volume of DMSO vehicle throughout the intervention period; ② DCA group: treated with DCA at a final concentration of 0.1 mM for the full 96 h; ③ DCA + IPA group: pre-incubated with 0.1 mM DCA for the first 24 h, then IPA was added to the medium at a final concentration of 0.1 mM, followed by co-incubation of DCA and IPA for the remaining 72 h until the end of the 96 h intervention. The final concentration of DMSO was consistent across all groups to eliminate solvent interference, and the drug-containing medium was renewed every 24 h during the intervention. The intervention concentrations and treatment schedule were determined with reference to the corresponding HT-29 cell-based conditions and were further optimized for the organoid culture system.

For morphological observation, bright-field images of organoids in each group were captured using an inverted microscope every 24 h, to record the growth status, budding rate, diameter and morphological changes of organoids. All analyses were performed based on the *in vitro* live-cell imaging results.

### 2.16 Fecal total DNA extraction

Total fecal DNA was extracted using the E.Z.N.A.^®^ Stool DNA Kit (D4015, Omega Bio-tek, USA) following the manufacturer’s standard protocol. Briefly, 100 mg of fresh fecal sample was homogenized with 200 mg Glass Beads X and 540 μL SLX-Mlus Buffer by vortexing at maximum speed for 10 min. After adding 60 μL DS Buffer and 20 μL Proteinase K Solution, the mixture was incubated at 70 °C for 10 min with intermittent vortexing. Then 200 μL SP2 Buffer was added, followed by ice incubation for 5 min and centrifugation at 13,000×g for 5 min. The supernatant was transferred and mixed with 200 μL fully resuspended cHTR Reagent, incubated at room temperature for 2 min, and centrifuged at 13,000×g for 2 min. The supernatant was then mixed with equal volumes of BL Buffer and absolute ethanol, and loaded onto HiBind^®^ DNA Mini Columns. After sequential washes with VHB Buffer and DNA Wash Buffer, the columns were centrifuged at 13,000×g for 2 min to remove residual ethanol. DNA was eluted with 100 μL preheated (65 °C) Elution Buffer and stored at -20 °C for subsequent qPCR analysis. The relative abundance of total fecal bacteria and *Clostridium cluster I* was determined by qPCR following the same protocol as described in Section 2.13. The primer sequences were as follows: Total fecal bacteria forward, TCCTACGGGAGGCAGCAGT; reverse, GGACTACCAGGGTATCTAATCCTGTT; *Clostridium cluster I* forward, TACCHRAGGAGGAAGCCAC; reverse, GTTCTTCCTAATCTCTACGCAT.

### 2.17 Statistical analysis

All data were expressed as mean ± SEM unless otherwise specified. Exact sample sizes (n) and the statistical tests used for each analysis are provided in the corresponding figure legends. Statistical analysis was performed using GraphPad Prism 10.0, with R 4.3.2 and SIMCA-P 14.1 for metabolomic and microbiome data analysis. For normally distributed data, unpaired t-test (two groups) or one-way ANOVA (multiple groups) was used. For non-normally distributed data, the Mann-Whitney U test (two groups) or Kruskal-Wallis test (multiple groups) was applied. Spearman correlation was used for variable association analysis. For some comparative plots showing relative changes across groups, only group mean values were presented to facilitate visual comparison. In targeted tryptophan metabolomics, selected metabolites were log10-transformed prior to visualization and statistical analysis when necessary to reduce heteroscedasticity and improve comparability between groups. Differences with p < 0.05 were considered statistically significant.

## 3 Results

### 3.1 DCA induces anxiety- and depression-like behaviors in mice similar to those triggered by HFD

**Figure 1:**
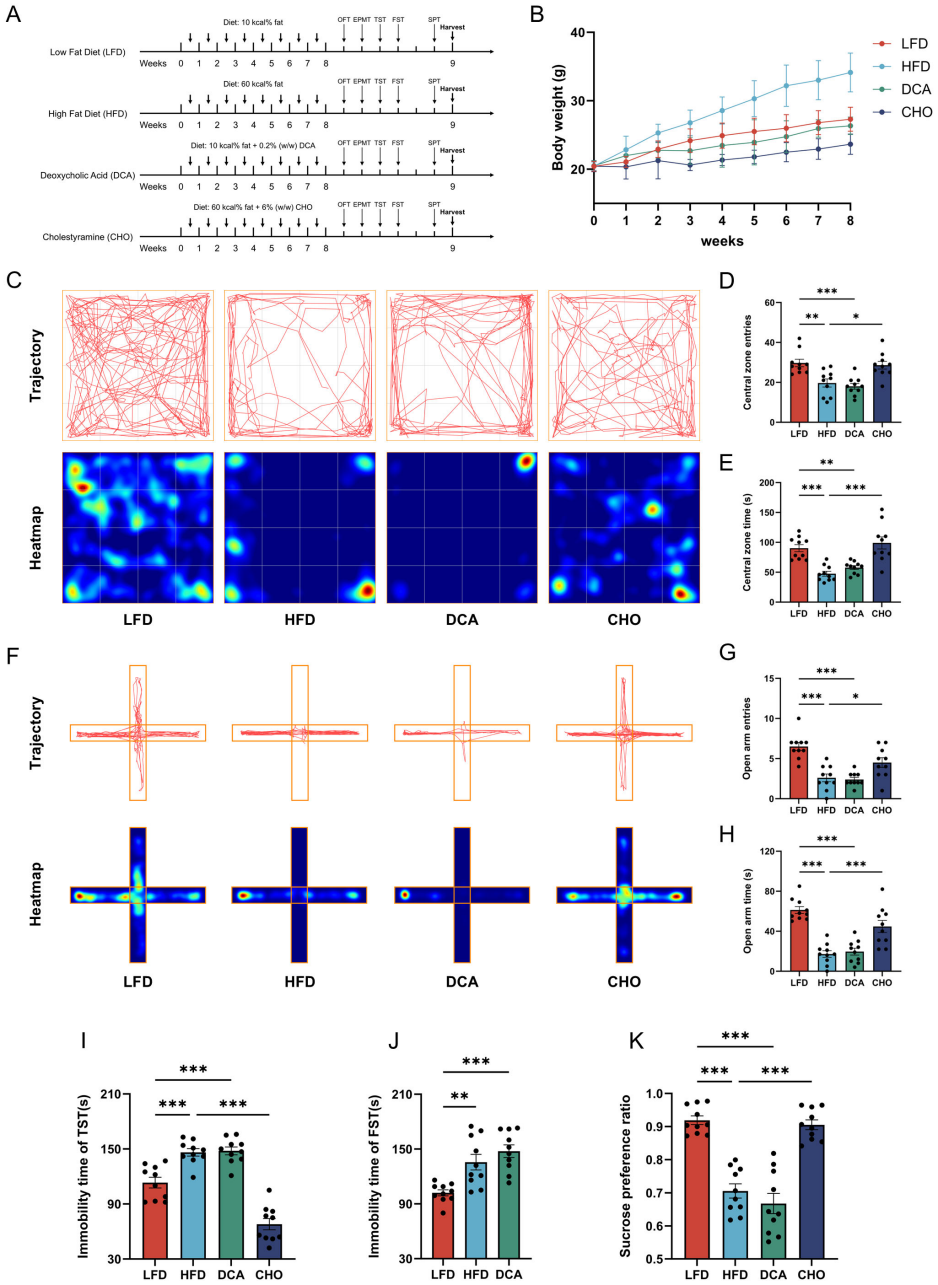
DCA induces anxiety- and depression-like behaviors in mice similar to those triggered by HFD. **(A)** Experimental design and intervention timeline. **(B)** Weekly body weight changes of mice in each group (n=10). **(C–E)** Representative trajectories/heatmaps **(C)**, and quantification of central area entries **(D)** (LFD vs HFD: p = 0.008, LFD vs DCA: p < 0.001, HFD vs CHO: p = 0.02, Kruskal-Wallis test) and stay duration **(E)** (LFD vs HFD: p < 0.001, LFD vs DCA: p = 0.003, HFD vs CHO: p < 0.001, one-way ANOVA) in OFT (n=10). **(F–H)** Representative trajectories/heatmaps **(F)**, and quantification of open arm entries **(G)** (LFD vs HFD: p < 0.001, LFD vs DCA: p < 0.001, HFD vs CHO: p = 0.02, one-way ANOVA) and stay duration **(H)** (LFD vs HFD: p < 0.001, LFD vs DCA: p < 0.001, HFD vs CHO: p < 0.001, one-way ANOVA) in EPMT (n=10). **(I–K)** Quantification of immobility time in TST **(I)** (LFD vs HFD: p < 0.001, LFD vs DCA: p < 0.001, HFD vs CHO: p < 0.001, one-way ANOVA) and FST **(J)** (CHO group not tested, LFD vs HFD: p = 0.003, LFD vs DCA: p < 0.001, one-way ANOVA), and sucrose preference ratio **(K)** (LFD vs HFD: p < 0.001, LFD vs DCA: p < 0.001, HFD vs CHO: p < 0.001, one-way ANOVA) (n=10). LFD, low fat diet; HFD, high fat diet; DCA, deoxycholic acid; CHO, cholestyramine; OFT, Open Field Test; EPMT, Elevated Plus Maze Test; TST, Tail Suspension Test; FST, Forced Swimming Test; SPT, Sucrose Preference Test. Data are presented as mean ± SEM. *p < 0.05, **p < 0.01, ***p < 0.001.

Our previous experiments have shown that 2 and 8 weeks of high-fat feeding led to elevated fecal DCA levels, accompanied by intestinal barrier dysfunction (14, 15). To further verify whether HFD-induced DCA elevation was sufficient to drive abnormal emotional behaviors, we established four mouse models with different dietary interventions and performed behavioral tests after 8 weeks (**Figure 1A**). Body weight monitoring results are presented in the figure (**Figure 1B**; **Supplementary Figure 1A**).

For anxiety-like behaviors, OFT trajectory and heatmap analyses showed clear differences among groups. Compared with the LFD group, mice in the HFD and DCA groups were more inclined to move along the periphery of the arena and exhibited reduced exploration of the central area (**Figure 1C**). Correspondingly, the number of entries into the central area and the total duration of stay in the central area were significantly lower in the DCA group than in the LFD group, with a trend consistent with that in the HFD group; concurrent CHO administration reversed these HFD-induced changes (**Figures 1D, E**). No obvious difference in total distance traveled was observed among groups in the OFT, suggesting that the reduced center exploration was not due to altered overall locomotor activity (**Supplementary Figure 1B**). Heatmaps and trajectory analyses of the EPMT similarly indicated that, compared with the LFD group, mice in the HFD and DCA groups showed reduced exploration of the vertical open arms and spent more time in the horizontal closed arms, and administration of CHO resulted in normalization of these HFD-induced behavior patterns (**Figure 1F**). Quantitative results showed that the number of entries into the open arms and the total duration of stay in the open arms were significantly decreased in the HFD and DCA groups compared with the LFD group, while concurrent CHO administration reversed these HFD-induced changes (**Figures 1G, H**). Total distance traveled also showed no obvious difference (**Supplementary Figure 1C**).

For depression-like behaviors, compared with the LFD group, the DCA group showed significantly prolonged immobility time in the TST and FST, as well as a significant reduction in sucrose preference, exhibiting a depression-like phenotype consistent with the HFD group (**Figures 1I–K**). CHO administration reversed the abnormal phenotypes in the TST and sucrose preference observed in the HFD group. The FST was not performed in the CHO group because these mice were unable to remain immobile while floating in water. This should be considered an assay-specific limitation, particularly because the other behavioral tests showed a consistent beneficial direction (**Figure 1J**).

These results suggest that DCA intervention induced anxiety- and depression-like behaviors in mice similar to those triggered by HFD.

### 3.2 DCA induces alterations in the gut microbiota and metabolites (IPA) of mice similar to those caused by HFD

**Figure 2:**
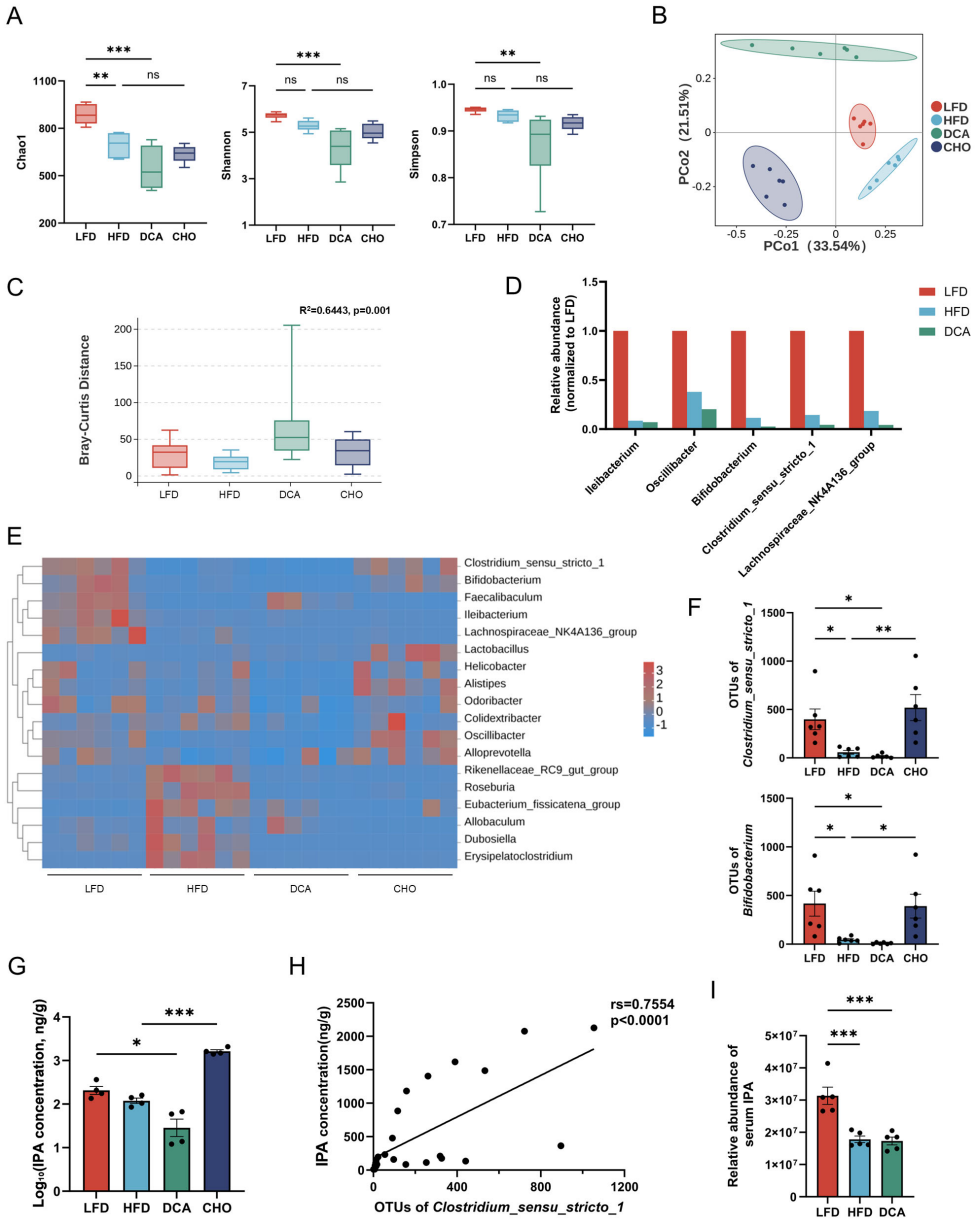
DCA induces alterations in the gut microbiota and metabolites (IPA) of mice similar to those caused by HFD. **(A)** Gut microbiota alpha diversity indices (Chao1, Shannon, Simpson) in each group (n=6). **(B–C)** Beta diversity analysis: PCoA plot **(B)** and Bray-Curtis distance **(C)** showing distinct microbiota structure among groups (n=6). **(D–F)** Differential bacterial genera analysis: relative abundance bar plot **(D)** (Data are presented as mean), clustered heatmap **(E)**, and OTU quantification of key genera *Clostridium_sensu_stricto_1* (LFD vs HFD: p = 0.03, LFD vs DCA: p = 0.02, HFD vs CHO: p = 0.004, one-way ANOVA) and *Bifidobacterium* (LFD vs HFD: p = 0.02, LFD vs DCA: p = 0.01, HFD vs CHO: p = 0.04, one-way ANOVA) **(F)** (n=6). **(G)** Fecal IPA concentration (log10-transformed) (LFD vs HFD: p = 0.20, LFD vs DCA: p = 0.04, HFD vs CHO: p < 0.001, one-way ANOVA) (n=4). **(H)** Spearman’s correlation between *Clostridium_sensu_stricto_1* abundance and fecal IPA concentration (rs = 0.7554, p < 0.0001, n=6). **(I)** Serum IPA relative abundance from untargeted metabolomics (LFD vs HFD: p < 0.001, LFD vs DCA: p < 0.001, one-way ANOVA) (n=5). LFD, low fat diet; HFD, high-fat diet; DCA, deoxycholic acid; CHO, cholestyramine; IPA, indole-3-propionic acid; PCoA, principal coordinates analysis; OTU, operational taxonomic unit. Data are presented as mean ± SEM unless other stated. *p < 0.05, **p < 0.01, ***p < 0.001.

Previous studies have shown that high concentrations of DCA can significantly inhibit the growth of multiple gut bacterial species (12). To explore the role of the gut microbiota and its metabolites in DCA-induced anxiety- and depression-like behaviors, we performed 16S rRNA gene sequencing on fecal samples from mice in each group. Compared with the LFD group, DCA intervention significantly reduced the Chao1, Shannon and Simpson alpha diversity indices of the gut microbiota in mice (**Figure 2A**). Beta diversity analysis showed clear separation of samples among the LFD, HFD, DCA, and CHO groups, suggesting significant differences in microbiota structure between groups (**Figures 2B, C**). At the genus level, multiple bacterial genera were decreased in both the HFD and DCA groups compared with the LFD group, including *Clostridium_sensu_stricto_1*, *Bifidobacterium*, *Oscillibacter*, and *Lachnospiraceae_NK4A136_group* (**Figure 2D**; **Supplementary Figures 2A, B**). A clustered heatmap further showed that *Clostridium_sensu_stricto_1* exhibited a changing pattern similar to that of *Bifidobacterium*. Compared with the LFD group, the abundance of these two genera was significantly reduced in the HFD and DCA groups, while CHO administration reversed the HFD-induced changes (**Figures 2E, F**).

Because compositional changes in the gut microbiota may be accompanied by altered microbial metabolite output, we next quantified predefined representative indole metabolites of the tryptophan pathway in fecal samples. The results showed that fecal IPA (log10-transformed) was significantly decreased in the DCA group compared with the LFD group, while the downward trend observed in the HFD group was reversed by concurrent CHO feeding (**Figure 2G**). Effect size analysis further supported the magnitude of these changes: a large effect was observed for LFD vs. HFD, a stronger large effect for LFD vs. DCA, and an extremely strong effect for HFD vs. CHO (**Supplementary Figures 2C, D**). In contrast, although IAA and ILA also showed changes, their alteration trends were not consistent with the abundance changes of *Clostridium_sensu_stricto_1* or *Bifidobacterium* (**Supplementary Figures 2E, F**). Spearman’s rank correlation analysis revealed a highly significant positive correlation between the relative abundance of *Clostridium_sensu_stricto_1* and fecal IPA concentration (rs = 0.7554, p < 0.0001; **Figure 2H**), suggesting a close association between changes in this genus and alterations in fecal IPA levels.

To determine whether intestinal metabolic alterations were also reflected systemically, we further performed untargeted metabolomics analysis on serum samples from model mice. Principal component analysis showed that both HFD and DCA modeling caused a clear separation of the serum metabolic profile from that of the LFD group (**Supplementary Figure 2G**). After comparing differential metabolites between LFD–HFD and LFD–DCA, we found that IPA was detected among the differential metabolites in both comparisons. Notably, IPA was the only indole-related metabolite that consistently emerged as a shared differential metabolite in both comparisons, whereas IAA and ILA did not show the same pattern. In addition, both HFD and DCA caused a significant decrease in the relative abundance of serum IPA (**Figure 2I**; **Supplementary Figures 2H, I**).

Taken together, these results showed that DCA modeling induced gut microbiota compositional changes accompanied by reduced fecal and serum IPA levels, which were broadly consistent with the characteristics exhibited by the HFD model.

### 3.3 DCA induces anxiety- and depression-like behaviors in mice via downregulating IPA levels

**Figure 3:**
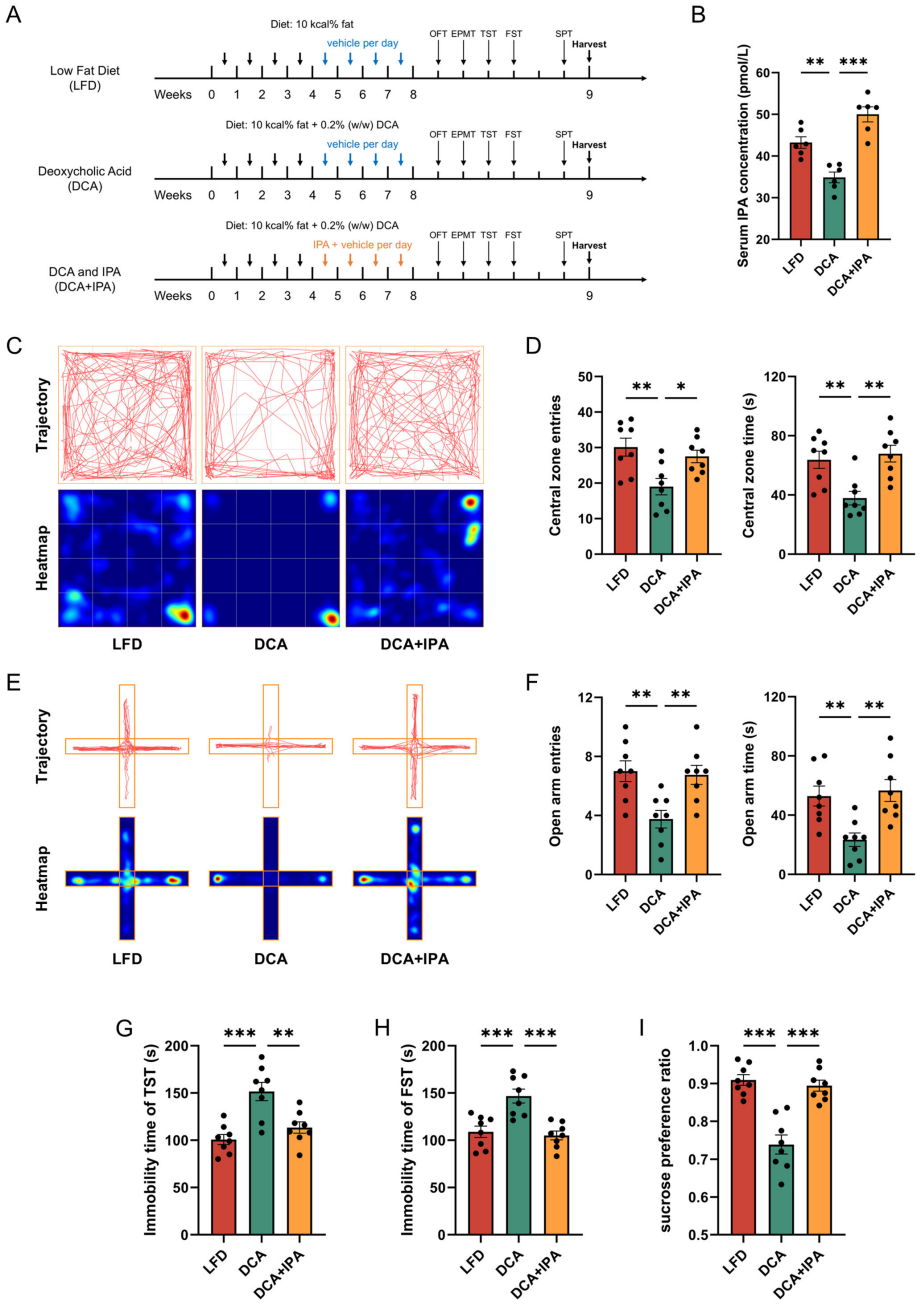
DCA induces anxiety- and depression-like behaviors in mice via downregulating IPA level. **(A)** Experimental design and timeline of 8-week DCA modeling, IPA intragastric intervention and behavioral test procedures. **(B)** Serum IPA concentration in LFD, DCA and DCA+IPA groups (LFD vs DCA: p = 0.003, DCA vs DCA+IPA: p < 0.001, one-way ANOVA) (n=6). **(C–D)** Representative movement trajectories/heatmaps **(C)**, and quantification of central area entries (LFD vs DCA: p = 0.004, DCA vs DCA+IPA: p = 0.03, one-way ANOVA) and total stay duration (LFD vs DCA: p = 0.005, DCA vs DCA+IPA: p = 0.002, one-way ANOVA) **(D)** in OFT (n=8). **(E–F)** Representative movement trajectories/heatmaps **(E)**, and quantification of open arm entries (LFD vs DCA: p = 0.004, DCA vs DCA+IPA: p = 0.007, one-way ANOVA) and total stay duration (LFD vs DCA: p = 0.007, DCA vs DCA+IPA: p = 0.003, one-way ANOVA) **(F)** in EPMT (n=8). **(G–I)** Quantification of immobility time in TST (LFD vs DCA: p < 0.001, DCA vs DCA+IPA: p = 0.003, one-way ANOVA) **(G)** and FST (LFD vs DCA: p < 0.001, DCA vs DCA+IPA: p < 0.001, one-way ANOVA) **(H)**, and sucrose preference ratio (LFD vs DCA: p < 0.001, DCA vs DCA+IPA: p < 0.001, one-way ANOVA) **(I)** (n=8). LFD, low fat diet; DCA, deoxycholic acid; IPA, indole-3-propionic acid; OFT, Open Field Test; EPMT, Elevated Plus Maze Test; TST, Tail Suspension Test; FST, Forced Swimming Test; SPT, Sucrose Preference Test. Data are presented as mean ± SEM. *p < 0.05, **p < 0.01, ***p < 0.001.

To investigate the association between DCA-induced reduction of IPA levels and anxiety- and depression-like behaviors, we performed oral IPA supplementation during weeks 5–8 of the 8-week DCA modeling protocol and conducted behavioral tests after 8 weeks (**Figure 3A**). Quantitative measurement of serum IPA confirmed that intragastric IPA supplementation markedly reversed the DCA-induced decrease in serum IPA levels (**Figure 3B**).

For anxiety-like behaviors, OFT results showed that, compared with the DCA group, mice in the IPA supplementation group had a significantly higher number of entries into the central area and a significantly longer duration of stay in the central area (**Figures 3C, D**). In the EPMT, the number of entries into the open arms and the duration of stay in the open arms were also significantly increased in the IPA supplementation group (**Figures 3E, F**). For depression-like behaviors, compared with the DCA group, mice in the IPA supplementation group exhibited shorter immobility time in the TST and FST, and sucrose preference was also markedly restored (**Figures 3G–I**).

These results suggest that IPA supplementation alleviated DCA-induced anxiety- and depression-like behaviors, indicating that reduced IPA levels may contribute to behavioral changes in DCA model mice.

### 3.4 IPA administration restores DCA-induced cerebral pathological changes through PXR pathways

**Figure 4:**
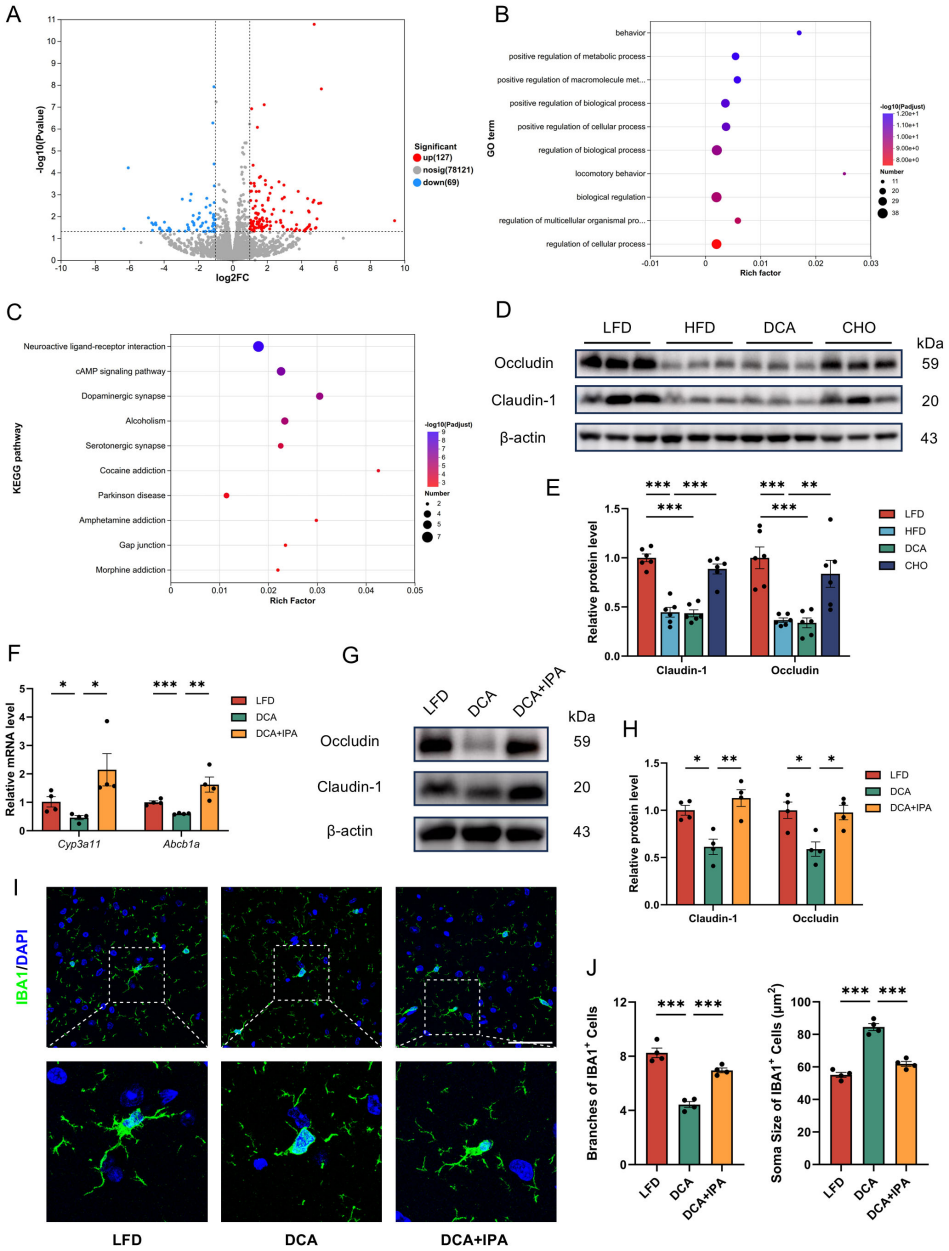
IPA administration restores DCA-induced cerebral pathological changes. **(A)** Volcano plot of differentially expressed genes from whole-brain transcriptome sequencing in LFD and DCA groups (n=3). **(B–C)** GO biological process enrichment **(B)** and KEGG pathway enrichment analysis **(C)** of the differentially expressed genes (n=3). **(D–E)** Western blot bands and quantitative analysis of barrier tight junction proteins Occludin (LFD vs HFD: p < 0.001, LFD vs DCA: p < 0.001, HFD vs CHO: p = 0.005, one-way ANOVA), Claudin-1 (LFD vs HFD: p < 0.001, LFD vs DCA: p < 0.001, HFD vs CHO: p < 0.001, one-way ANOVA) in LFD, HFD, DCA and CHO groups (n=6). **(F)** Relative mRNA levels of *Cyp3a11* (LFD vs DCA: p = 0.03, DCA vs DCA+IPA: p = 0.03, Kruskal-Wallis test) and *Abcb1a* (LFD vs DCA: p < 0.001, DCA vs DCA+IPA: p = 0.008, one-way ANOVA) in mouse brain tissue across the LFD, DCA and DCA+IPA groups (n=4). **(G–H)** Western blot bands and quantitative analysis of brain tight junction proteins Occludin (LFD vs DCA: p = 0.01, DCA vs DCA+IPA: p = 0.02, one-way ANOVA), Claudin-1 (LFD vs DCA: p = 0.01, DCA vs DCA+IPA: p = 0.002, one-way ANOVA) in LFD, DCA and DCA+IPA groups (n=4). **(I–J)** Representative IBA1 immunofluorescence images **(I)** and quantitative analysis of microglial branch number (LFD vs DCA: p < 0.001, DCA vs DCA+IPA: p < 0.001, one-way ANOVA) and soma area (LFD vs DCA: p < 0.001, DCA vs DCA+IPA: p < 0.001, one-way ANOVA) **(J)** in each group (n=4). Scale bar, 50 μm. LFD, low fat diet; HFD, high-fat diet; DCA, deoxycholic acid; CHO, cholestyramine; IPA, indole-3-propionic acid; GO, Gene Ontology; KEGG, Kyoto Encyclopedia of Genes and Genomes; IBA1, Ionized calcium-binding adapter molecule 1. Data are presented as mean ± SEM. *p < 0.05, **p < 0.01, ***p < 0.001.

To explore the potential mechanisms underlying the effects of DCA on cerebral function, we performed whole-brain transcriptome sequencing on DCA model mice and LFD mice. Volcano plot analysis showed that, compared with the LFD group, 127 genes were significantly upregulated and 69 genes were significantly downregulated in the DCA group (**Figure 4A**). Gene Ontology (GO) enrichment analysis indicated that the differentially expressed genes were significantly enriched in neural behavior-related terms, including behavior, locomotor behavior, and biological regulation, and were also enriched in biological processes related to cellular stress and injury responses, such as regulation of metabolic processes and regulation of cellular processes (**Figure 4B**). Kyoto Encyclopedia of Genes and Genomes (KEGG) enrichment analysis further showed that the differentially expressed genes were significantly enriched in key neural signaling pathways, including neuroactive ligand-receptor interaction, cAMP signaling pathway, dopaminergic synapse, and serotonergic synapse, and were also enriched in pathways related to cellular injury repair and inflammation regulation, such as autophagy and gap junction (**Figure 4C**). These results suggest that DCA modeling induced marked transcriptomic alterations in the brain. Given the enrichment of the dopaminergic synapse pathway, we next examined representative dopamine signaling-related genes associated with emotional behavior. qPCR validation further confirmed increased *Drd2* expression and decreased *Fos* expression in the DCA group, supporting the involvement of altered neural signaling activity in this model (**Supplementary Figure 3A**).

Considering that changes in peripheral metabolism and inflammatory status may affect brain barrier homeostasis, we further assessed the expression of tight junction proteins related to the mechanical barrier of the brain. Western blot results showed that Claudin-1 and Occludin were significantly downregulated in the HFD and DCA groups compared with the LFD group, while CHO intervention reversed the HFD-induced decrease (**Figures 4D, E**), indicating that DCA modeling mimicked HFD-induced impairment of brain barrier function. After IPA supplementation in DCA model mice, the expression of genes *Cyp3a11* and *Abcb1a* downstream of PXR were significantly increased, as well as the restored expression levels of Claudin-1 and Occludin, which were consistent with activation of IPA-PXR-related protective signaling. (**Figures 4F–H**), suggesting that DCA-induced downregulation of brain barrier-related proteins may be associated with IPA depletion. In addition, we evaluated microglial activation status. IBA1 immunofluorescence staining and morphological quantitative analysis showed that, compared with the LFD group, DCA modeling induced a significant reduction in the number of microglial branches and a marked increase in soma area, similar to changes observed with HFD, indicating functional activation of microglia; CHO intervention reversed these HFD-induced changes (**Supplementary Figures 3B, C**). After IPA supplementation in DCA model mice, microglial morphology was markedly restored (**Figures 4I, J**).

Taken together, these results indicate that DCA modeling was associated with cerebral pathological changes, including brain transcriptomic alterations, downregulation of PXR-related signaling and brain barrier-related proteins, and microglial activation, while IPA supplementation exerted a marked reversal effect on these cerebral pathological phenotypes, providing further evidence that reduced IPA levels may contribute to DCA-related behavioral abnormalities.

### 3.5 IPA administration restores DCA-induced intestinal barrier dysfunction through PXR pathways

**Figure 5:**
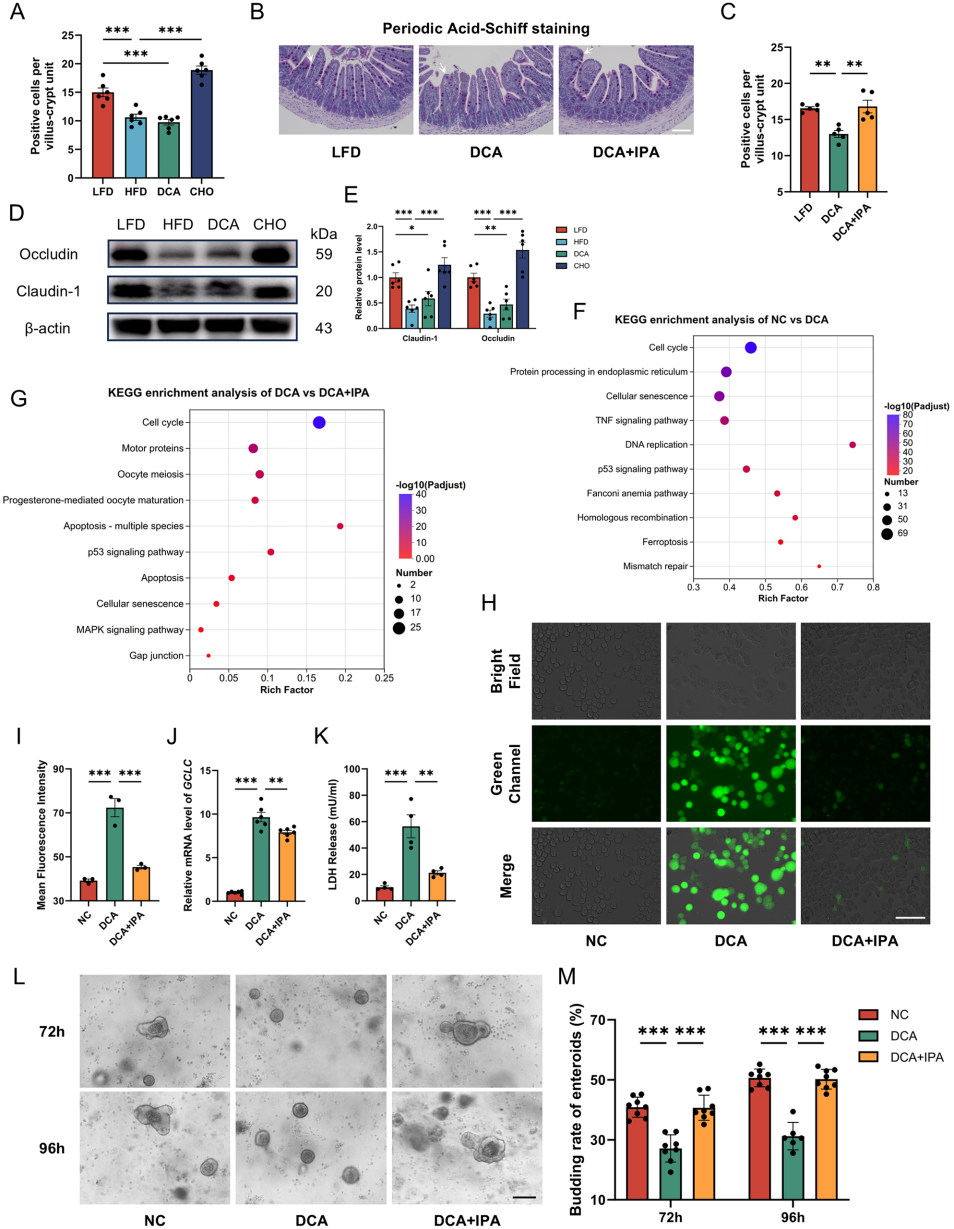
IPA administration restores DCA-induced intestinal barrier dysfunction. **(A)** Quantitative analysis of ileal goblet cells (PAS staining) per villus-crypt unit in LFD, HFD, DCA and CHO groups (LFD vs HFD: p < 0.001, LFD vs DCA: p < 0.001, HFD vs CHO: p < 0.001, one-way ANOVA) (n=6). **(B–C)** Representative PAS staining images of the ileum **(B)** and goblet cell quantification (LFD vs DCA: p = 0.002, DCA vs DCA+IPA: p = 0.001, one-way ANOVA) **(C)** in LFD, DCA and DCA+IPA groups (n=5). Scale bar, 100 μm. **(D–E)** Western blot bands and quantitative analysis of intestinal tight junction proteins Occludin (LFD vs HFD: p < 0.001, LFD vs DCA: p = 0.007, HFD vs CHO: p < 0.001, one-way ANOVA), Claudin-1 (LFD vs HFD: p < 0.001, LFD vs DCA: p = 0.03, HFD vs CHO: p < 0.001, Kruskal-Wallis test) in LFD, HFD, DCA and CHO groups (n=6). **(F–G)** KEGG pathway enrichment analysis of differentially expressed genes from HT-29 cell transcriptome sequencing: NC vs DCA **(F)**, DCA vs DCA+IPA **(G)** (n=4). **(H–I)** ROS detection in HT-29 cells: representative fluorescence images **(H)**, mean fluorescence intensity quantification (NC vs DCA: p < 0.001, DCA vs DCA+IPA: p < 0.001, one-way ANOVA) **(I)** (n=3). Scale bar, 75 μm. **(J)** *GCLC* mRNA expression level in HT-29 cells (NC vs DCA: p < 0.001, DCA vs DCA+IPA: p = 0.005, one-way ANOVA) (n=6). **(K)** LDH release level in HT-29 cells (NC vs DCA: p < 0.001, DCA vs DCA+IPA: p = 0.002, one-way ANOVA) (n=4). **(L–M)** Representative bright-field images of mouse ileal organoids **(L)** and quantitative analysis of organoid budding rate at 72 h (NC vs DCA: p < 0.001, DCA vs DCA+IPA: p < 0.001, one-way ANOVA) and 96 h (NC vs DCA: p < 0.001, DCA vs DCA+IPA: p < 0.001, one-way ANOVA) **(M)** (n=6-8). Scale bar, 100 μm. LFD, low fat diet; HFD, high fat diet; DCA, deoxycholic acid; CHO, cholestyramine; NC, normal control; IPA, indole-3-propionic acid; PAS, Periodic Acid-Schiff; ROS, reactive oxygen species; LDH, lactate dehydrogenase; KEGG, Kyoto Encyclopedia of Genes and Genomes. Data are presented as mean ± SEM. *p < 0.05, **p < 0.01, ***p < 0.001.

The above findings supported the brain functional basis of IPA-mediated effects of DCA modeling on anxiety- and depression-like behaviors. As a gut microbiota-derived metabolite, we next examined whether changes in intestinal IPA were also associated with intestinal function. At the level of the intestinal mucus barrier, the number of goblet cells was decreased in both the HFD and DCA groups compared with the LFD group, while concurrent CHO administration restored the reduced number of goblet cell caused by the HFD (**Figure 5A**; **Supplementary Figure 4A**). IPA supplementation significantly reversed the DCA-induced reduction in goblet cell numbers (**Figures 5B, C**). For the intestinal mechanical barrier, Western blot results showed that the expression levels of the tight junction proteins Claudin-1 and Occludin were significantly decreased in the DCA group, consistent with the trend in the HFD group, while CHO intervention reversed the HFD-induced changes (**Figures 5D, E**). To further dissect the potential mechanisms underlying DCA-induced injury and the reversal effect of IPA supplementation, we performed transcriptome sequencing in cell intervention models. The results showed significant differences in gene expression profiles among the three cell groups (**Supplementary Figure 4B**). KEGG pathway enrichment analysis showed that, compared with the NC group, differentially expressed genes in the DCA group were significantly enriched in pathways closely related to cellular injury and stress responses, including cell cycle, protein processing in endoplasmic reticulum, cellular senescence, and ferroptosis, suggesting that DCA treatment broadly activated intracellular injury signaling networks (**Figure 5F**). In the comparison between the DCA + IPA group and the DCA group, differentially expressed genes were significantly enriched in core regulatory pathways including cell cycle, p53 signaling pathway, and cellular senescence, accompanied by enrichment of pathways related to cellular structural repair such as motor proteins and gap junctions, indicating that IPA supplementation may reverse DCA-induced abnormal signaling regulation (**Figure 5G**). These results indicated a potential marked change in cellular injury. qPCR validation further showed that, compared with the DCA group, IPA supplementation increased the expression of *CYP3A4* and *ABCB1*, suggesting the activation of PXR-related protective signaling (**Supplementary Figure 4C**).

We further assessed downstream injury phenotypes. Oxidative stress assays showed that DCA treatment induced substantial ROS production and significantly upregulated the expression of oxidative stress-related genes, and these changes were reversed by IPA supplementation (**Figures 5H–J**). LDH release assays showed that LDH release levels were significantly increased in the DCA group and were significantly reduced after IPA supplementation (**Figure 5K**). In addition, we performed DCA and DCA + IPA interventions in mouse ileal organoids. Compared with the NC group, organoid budding efficiency was significantly reduced in the DCA group after 72 h and 96 h of intervention, while IPA supplementation significantly restored budding efficiency (**Figures 5L, M**).

Taken together, these results indicate that DCA can induce intestinal barrier injury by inducing oxidative stress in intestinal epithelial cells, while the supplementation of IPA effectively alleviated this damaging effect. The protective effect of IPA was accompanied by restoration of barrier-related phenotypes, reduction of oxidative injury, and activation of PXR signaling.

### 3.6 FMT intervention replicates the anxiety- and depression-like behaviors observed in mice caused by DCA

**Figure 6:**
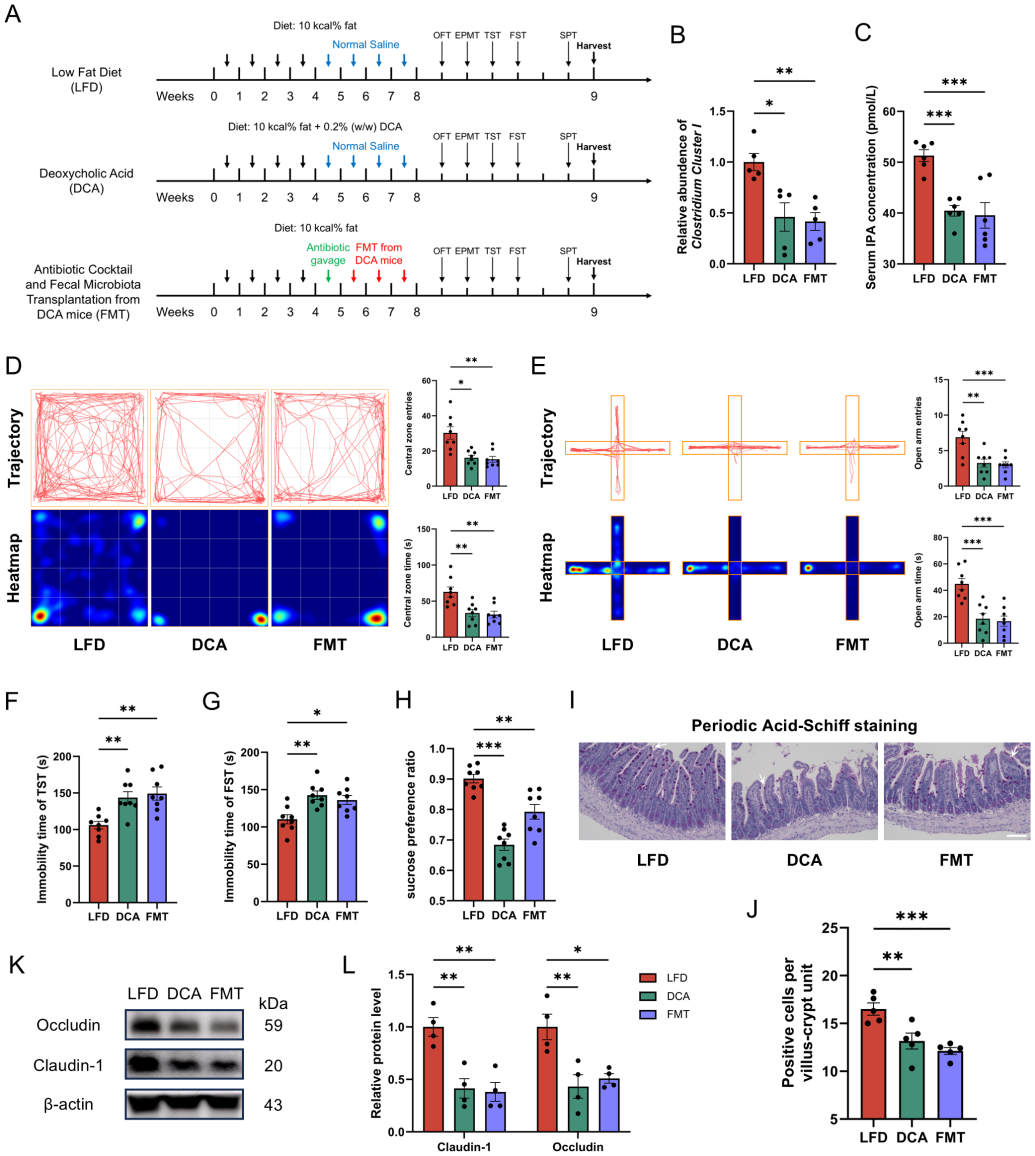
FMT intervention replicates the anxiety- and depression-like behaviors observed in mice caused by DCA. **(A)** Experimental design and timeline of 8-week DCA modeling, antibiotic cocktail pretreatment, FMT intervention and sequential behavioral test procedures. **(B)** Relative abundance of *Clostridium cluster I* in fecal microbiota of LFD, DCA, and FMT groups (LFD vs DCA: p = 0.03, LFD vs FMT: p = 0.009, Kruskal-Wallis test) (n=5). **(C)** Serum IPA concentration in LFD, DCA and FMT groups (LFD vs DCA: p < 0.001, LFD vs FMT: p < 0.001, one-way ANOVA) (n=6). **(D)** Representative movement trajectories/heatmaps and quantification of central area entries (LFD vs DCA: p = 0.01, LFD vs FMT: p = 0.003, Kruskal-Wallis test) and total stay duration (LFD vs DCA: p = 0.003, LFD vs FMT: p = 0.001, one-way ANOVA) in OFT (n=8). **(E)** Representative movement trajectories/heatmaps and quantification of open arm entries (LFD vs DCA: p = 0.001, LFD vs FMT: p < 0.001, one-way ANOVA) and total stay duration (LFD vs DCA: p < 0.001, LFD vs FMT: p < 0.001, one-way ANOVA) in EPMT (n=8). **(F–H)** Quantification of immobility time in TST (LFD vs DCA: p = 0.004, LFD vs FMT: p = 0.001, one-way ANOVA) **(F)** and FST (LFD vs DCA: p = 0.002, LFD vs FMT: p = 0.01, one-way ANOVA) **(G)**, and sucrose preference ratio (LFD vs DCA: p < 0.001, LFD vs FMT: p = 0.001, one-way ANOVA) **(H)** (n=8). **(I–J)** Representative PAS staining images of the ileum **(I)**, and quantification of ileal goblet cells per villus-crypt unit (LFD vs DCA: p = 0.006, LFD vs FMT: p < 0.001, one-way ANOVA) **(J)** (n=5). Scale bar, 100 μm. **(K–L)** Western blot bands **(K)** and quantitative analysis of the brain tight junction proteins Occludin (LFD vs DCA: p = 0.006, LFD vs FMT: p = 0.01, one-way ANOVA) and Claudin-1 (LFD vs DCA: p = 0.003, LFD vs FMT: p = 0.002, one-way ANOVA) **(L)** (n=4). LFD, low fat diet; HFD, high fat diet; DCA, deoxycholic acid; CHO, cholestyramine; IPA, indole-3-propionic acid; FMT, fecal microbiota transplantation; OFT, Open Field Test; EPMT, Elevated Plus Maze Test; TST, Tail Suspension Test; FST, Forced Swimming Test; SPT, Sucrose Preference Test; PAS, Periodic Acid-Schiff. Data are presented as mean ± SEM. *p < 0.05, **p < 0.01, ***p < 0.001.

To investigate the association between gut microbiota composition and anxiety- and depression-like behaviors, we transplanted fecal microbiota from DCA model mice into control mice and conducted behavioral tests after 8 weeks (**Figure 6A**). As a limited assessment of post-transplant microbial transfer, qPCR analysis showed that the relative abundance of *Clostridium cluster I* in FMT recipient mice was reduced, consistent with the change observed in DCA model mice (**Figure 6B**). These findings provided partial support for the transfer of donor-associated microbial features. Serum IPA levels in the FMT group were significantly decreased, similar to the changes observed in the DCA group, compared with the LFD group (**Figure 6C**).

OFT results showed that, compared with the LFD group, the number of entries into the central area and the duration of stay in the central area were significantly reduced in the FMT group (**Figure 6D**). In the EPMT, the number of entries into the open arms and the duration of stay in the open arms were also significantly decreased in the FMT group (**Figure 6E**). For depression-like behaviors, compared with the LFD group, mice in the FMT group exhibited longer immobility time in the TST and FST, as well as a significant reduction in sucrose preference (**Figures 6F–H**). These results suggest that FMT induced anxiety- and depression-like behaviors similar to those induced by DCA modeling, indicating that microbiota alterations may be a key factor mediating behavioral changes in DCA model mice.

Examination of intestinal and related indicators in FMT recipient mice revealed that the number of goblet cells in the terminal ileum was significantly reduced compared with the LFD group, a phenotype consistent with that in DCA model mice (**Figures 6I, J**). In addition, the expression levels of the tight junction proteins Claudin-1 and Occludin in brain were significantly downregulated in the FMT group (**Figures 6K, L**), and the number of branches and soma area of microglia also showed activation changes consistent with those in DCA model mice (**Supplementary Figures 5A, B**).

Taken together, these results support that DCA modeling-induced gut microbiota alterations can drive the development of anxiety- and depression-like behaviors in mice. DCA induces microbiota dysbiosis, which in turn impairs the intestinal barrier, reduces serum IPA levels, and ultimately leads to cerebral pathological changes and corresponding anxiety- and depression-like behavioral abnormalities.

## 4 Discussion

In this study, we revealed that exogenous supplementation with DCA can result in the replication of HFD-related anxiety- and depression-like behavioral phenotypes in an LFD background, whereas reducing the luminal DCA load via the bile acid binder CHO markedly reversed HFD-induced emotional and behavioral abnormalities. Exogenous supplementation with IPA, which was reduced by a HFD or DCA, significantly reversed the emotional and behavioral abnormalities induced by DCA. These results help elucidate how a HFD can interact with gut–brain signaling to promote emotional dysfunction.

Numerous studies have reported an association between a HFD and emotional disorders (3–5, 34, 35), but the peripheral factors driving these behavioral abnormalities remain unclear. Previous studies of DCA have focused mainly on its role in liver diseases, intestinal inflammation, colorectal cancer, and other disorders (36–38), whereas its effects on CNS function remain underexplored. Notably, a recent mouse study revealed that gut microbiota-derived DCA contributes to dietary cholesterol-associated cognitive impairment in individuals with obesity, but whether DCA also contributes to anxiety- and depression-related phenotypes has remained unclear (17). The central finding of this study is that the abnormal accumulation of DCA induced by a HFD is sufficient to drive the development of anxiety- and depression-like behaviors rather than representing a byproduct of obesity or other metabolic disorders. Conversely, reducing the luminal bile acid burden with CHO supplementation ameliorated HFD-associated behavioral abnormalities across the OFT, EPMT, TST, and SPT, further supporting the functional relevance of excessive intestinal DCA exposure. Therefore, the present behavioral findings support that HFD-associated DCA accumulation may be functionally relevant to the emergence of anxiety- and depression-like behaviors in mice.

After identifying DCA as an upstream factor, we further explored the possible downstream microbiota and metabolite mechanisms. Our data showed that DCA intervention significantly reduced alpha diversity and reshaped beta diversity. At the genus level, *Clostridium_sensu_stricto_1* was among the taxa whose abundance consistently changed after both HFD and DCA intervention. Our previous work revealed that CHO reversed the HFD-induced increase in DCA (14, 15), and in this study, we observed that CHO markedly reversed the HFD-induced reduction in the abundance of this genus, suggesting that its abundance is associated with intestinal DCA concentrations. Previous studies have indicated that certain members of *Clostridium_sensu_stricto_1* are key gut genera involved in tryptophan metabolism and the production of indole derivatives. Among them, strains such as *Clostridium sporogenes* can convert tryptophan into multiple indole metabolites, including IPA, IAA, and ILA (39, 40). Importantly, metabolomics analyses in the present study revealed that DCA intervention led to a synchronous and significant decrease in IPA levels in mouse feces and serum and that the abundance of *Clostridium_sensu_stricto_1* was significantly positively correlated with the fecal IPA concentration, supporting a close link between this taxonomic shift and impaired intestinal IPA production. In contrast, other indole metabolites did not show trends consistent with changes in microbiota abundance or behavioral phenotypes, which strengthened the specificity of IPA as the metabolite most consistently linked to the DCA-associated phenotype in this dataset. Together, these results indicate that the HFD-induced elevation in DCA levels reduced the abundance of *Clostridium_sensu_stricto_1* and, in parallel, decreased IPA levels in the intestine and circulation, suggesting a potential causal relationship among these alterations.

The tryptophan metabolic pathway has emerged as an important focus in gut–brain axis and emotional disorder research and is recognized as a central hub linking the gut microbiota, mucosal immune homeostasis, and CNS function via three main pathways: the kynurenine pathway, the 5-hydroxytryptamine pathway, and the indole pathway (41–43). Microbiota-derived indole metabolites can regulate host immune and barrier homeostasis and influence CNS function via the gut–brain axis, making them plausible downstream mediators of DCA-associated behavioral change (44, 45). Among these metabolites, IPA emerged as the most compelling candidate in our dataset because it showed the most consistent pattern across DCA exposure and microbiota-associated changes. IPA acts as an endogenous ligand for host PXR, which can upregulate tight junction protein expression in intestinal epithelial cells, enhance intestinal mucosal barrier integrity, and suppress intestinal inflammatory responses (22, 23). IPA has also been linked to antioxidant, anti-inflammatory, and neuroprotective effects (46). Although prior studies have described multiple biological functions of IPA, its role in HFD-related emotional disorders and its upstream regulatory mechanisms have not been systematically elucidated. Therefore, to determine whether reduced IPA is a necessary mediator of DCA-induced anxiety- and depression-like behaviors, we administered IPA by gavage to DCA model mice. The results showed that IPA gavage not only restored the DCA-induced decrease in serum IPA levels but also consistently reversed DCA-induced anxiety- and depression-like phenotypes. These supplementation–reversal rescue experiments support that the downregulation of IPA is functionally relevant to DCA-induced anxiety- and depression-like behaviors in mice. In addition, our IPA supplementation can mimic the function of gut microbiota in producing this endogenous metabolite, confirming the physiological relevance and improving the clinical translational potential of these findings.

After clarifying the reversal effect of IPA on anxiety- and depression-like behaviors, we further explored the mechanism underlying this effect from the perspective of the gut–brain axis. Whole-brain transcriptome sequencing revealed that genes whose expression was differentially expressed in response to DCA were significantly enriched in neural signaling pathways closely related to emotional regulation, including dopaminergic and serotonergic signaling, as well as pathways associated with cellular stress and injury responses. Among these, alterations in dopaminergic and serotonergic signaling are highly relevant to current neurobiological models of depression and remain important targets of antidepressant drugs (47–49). *Drd2*, a key receptor in dopaminergic signaling, is closely involved in neural processes relevant to motivation, reward, and emotional regulation (50). *Fos* is a classical immediate-early gene widely used as a marker of neuronal activation (51). Increased *Drd2* expression and decreased *Fos* expression in the DCA group may reflect reduced neural activity in behavior-related circuits. Together, these changes further support the presence of altered dopamine-related neural signaling. The BBB is a critical interface between the CNS and the periphery, and its integrity helps maintain central immune homeostasis (52). In this study, microbiota-derived IPA significantly reversed the DCA-induced downregulation of BBB-related proteins, supporting a role for IPA in maintaining barrier homeostasis. Notably, IPA supplementation also restored the expression of the PXR downstream genes *Cyp3a11* and *Abcb1a*, which is consistent with the activation of PXR-related protective signaling associated with vascular endothelial cells and the BBB (53, 54). In addition, a previous study demonstrated that IPA attenuated BBB injury and upregulated tight junction proteins through modulation of the PXR signaling pathway (55). Previous work has also indicated that the gut microbiota and its metabolites, such as indoles and short-chain fatty acids, can regulate cerebral vascular endothelial cells and maintain BBB integrity (56–58). Microglia are resident innate immune cells in the CNS, and excessive microglial activation is tightly linked to neuroinflammation, which has been widely implicated in anxiety and depression (59, 60). Here, DCA induced a change in microglial morphology characterized by reduced branching and enlarged soma, whereas IPA supplementation markedly reversed these changes, resulting in a more reactive state. These findings provide a plausible central neuroimmune correlate for the anxiolytic and antidepressant effects of IPA. Accordingly, we propose a working model in which the DCA-induced reduction in IPA may contribute to the downregulation of brain barrier-related proteins and a more reactive microglial state, thus promoting a cerebral environment associated with anxiety- and depression-like behaviors.

As an important initiating portal for signal transmission in the gut–brain axis, the intestinal barrier maintains immune homeostasis and prevents intestinal microorganisms and toxins from entering the circulation, and its structural and functional integrity is an important determinant of systemic inflammation levels (61, 62). Our previous study further suggested that intestinal barrier injury could drive the development of anxiety- and depression-like behaviors in mice by promoting the entry of lipopolysaccharides into the circulation (63). In this study, we observed a reduction in the number of goblet cells and a decrease in the expression of tight junction proteins in response to DCA, which is broadly consistent with the findings of our previous reports. Notably, these changes paralleled those observed in the HFD group, and both were reversed by the corresponding interventions of IPA or CHO. Zeng et al. reported that DCA increases intestinal epithelial permeability and disrupts intestinal barrier function by regulating the expression of cell junction-related genes in intestinal epithelial cells (13), which is in line with our observation of reduced Claudin-1 and Occludin expression. Our previous work revealed that DCA impairs intestinal stem cell function via the aryl hydrocarbon receptor pathway, leading to intestinal mucosal barrier dysfunction (14). In *in vitro* experiments, IPA reversed the DCA-induced increases in ROS production and LDH release in HT-29 cells, which is consistent with the antioxidant and cytoprotective properties of IPA. This interpretation was further supported by the results of transcriptome sequencing in the cell intervention model, in which DCA-enriched pathways related to cellular injury and stress responses were identified, whereas IPA supplementation was associated with the reversal of abnormal signaling pathways related to the cell cycle, p53 signaling, cellular senescence, and structural repair. In ileal organoids, DCA directly inhibited budding efficiency, which was restored by IPA supplementation. As organoid budding reflects epithelial growth and regenerative capacity, these results further support the direct effects of DCA and IPA on intestinal epithelial function. These findings indicate that IPA antagonized DCA-induced intestinal injury and supported a direct protective effect of IPA on intestinal epithelial cells in both cell and organoid models. Previous studies have reported that IPA can signal through PXR and restrain pro-inflammatory pathways to alleviate intestinal epithelial injury and enhance intestinal barrier homeostasis (23, 64). Consistent with this possibility, IPA supplementation in our cell model increased the expression of the PXR downstream genes *CYP3A4* and *ABCB1*. The above studies have provided promising mechanistic support for the protective effect of IPA on the intestinal barrier.

FMT is widely regarded as one of the most informative experimental approaches for testing microbiota causality and can provide strong evidence for a driving role of the microbiota in phenotype development. Previous studies have shown that transplantation of obesity-associated microbiota into germ-free mice induces obesity, glycolipid metabolic disorders, and intestinal barrier damage in recipient mice (65, 66). Kelly et al. transplanted the fecal microbiota from patients with depression into microbiota-depleted rats and induced depression-like behaviors in the recipients, providing causal evidence that microbiota dysbiosis contributes to depression-related phenotypes (67). To further assess whether alterations in the gut microbiota contribute to DCA-induced emotional and behavioral abnormalities, we performed FMT and transferred the fecal microbiota from DCA model mice to recipient mice after antibiotic-mediated microbiota depletion. Our data showed that, FMT alone induced anxiety- and depression-like behaviors in recipient mice that were similar to those observed in DCA model mice. The recipients also exhibited key pathological phenotypes consistent with those observed in the DCA model, including decreased serum IPA levels, a reduced number of ileal goblet cells, downregulation of brain tight junction proteins, and microglial morphological changes consistent with activation. These results provide strong additional support for the central hypothesis of this study and indicate that gut microbiota remodeling is an important mediator of the effects of DCA on anxiety- and depression-like behaviors. This finding underscores that bile acid-mediated gut microbiota dysregulation acts as a critical mediator of HFD-related emotional disorders.

This study still has several limitations. First, the current microbiota analysis was based mainly on genus-level 16S rRNA sequencing. Future studies should integrate metagenomics to identify key functional bacteria responsible for IPA production, obtain more direct causal evidence through single-strain supplementation, and explore the synergistic effects of combined interventions with functional bacteria and CHO to evaluate the potential of simultaneously regulating bile acid metabolism and microbiota function. Second, the specific mechanisms by which IPA exerts its protective effects remain unclear, and further research can be performed using approaches such as receptor antagonism and gene knockout. Third, the permeability of the intestinal and brain barriers requires further validation, and the key brain regions involved remain to be clarified; combining intestinal permeability tracers with brain region-specific sequencing may help refine the bile acid–microbiota–metabolite–barrier–CNS mechanistic chain. Finally, behavioral testing paradigms could be further optimized to reduce metabolic confounding, thus providing more precise parameter support for clinical translation.

## 5 Conclusions

**Figure 7:**
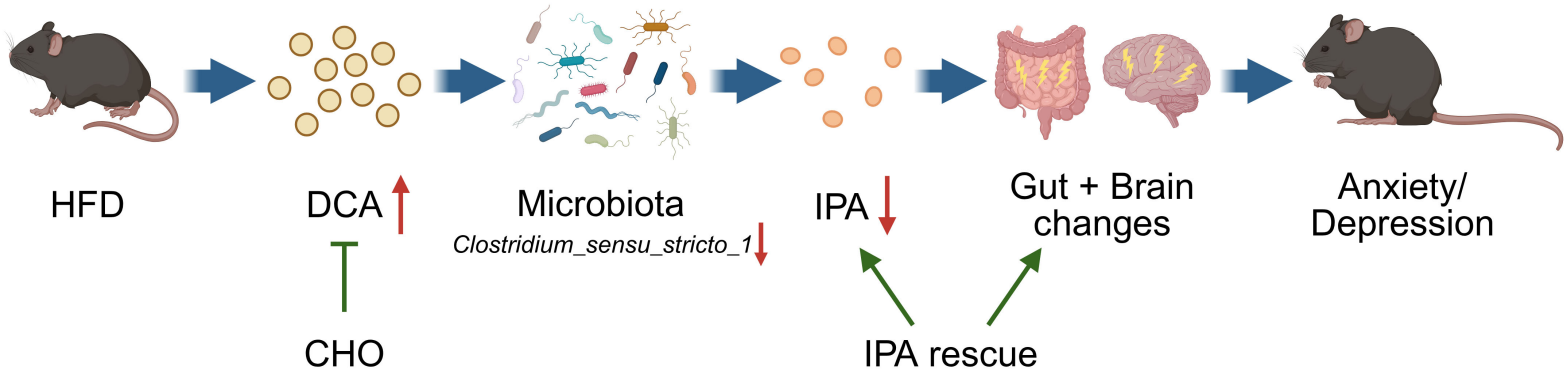
Mechanistic model of HFD-induced emotional disorders via the DCA–microbiota–IPA gut–brain axis. Schematic depicting the proposed pathway: HFD increases DCA, reduces *Clostridium_sensu_stricto_1* and IPA, leading to gut-brain dysfunction and anxiety/depression. Effects are rescued by CHO or IPA supplementation. HFD, high-fat diet; DCA, deoxycholic acid; CHO, cholestyramine; IPA, indole-3-propionic acid.

This study identified a potential gut–brain pathway linking HFD-associated luminal DCA elevation to anxiety- and depression-like behaviors in mice. In this model, the reduction in IPA resulting from DCA-induced microbiota remodeling appears to be a central mediator of DCA-driven regulation of emotional behaviors (**Figure 7**). These findings provide a basis for future studies to explore whether modulating bile acid metabolism or restoring IPA signaling could prevent or treat HFD-related emotional disorders.

## Data Availability

The datasets presented in this study can be found in online repositories. The raw data are available in the NCBI SRA database under the following BioProject accessions: PRJNA1464677, PRJNA1464653, and PRJNA1464583.
