# Supplementary material for: Deoxycholic acid promotes anxiety- and depression-like behaviors in mice via modulation of the gut microbial metabolite indole-3-propionic acid
Source: Front Immunol. 2026 Jun 11;17:1840574. doi: 10.3389/fimmu.2026.1840574 (PMC13294465; doi:10.3389/fimmu.2026.1840574)

Supplementary Figure 1

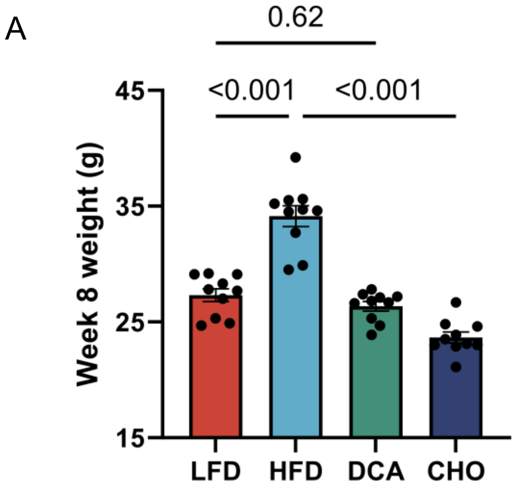

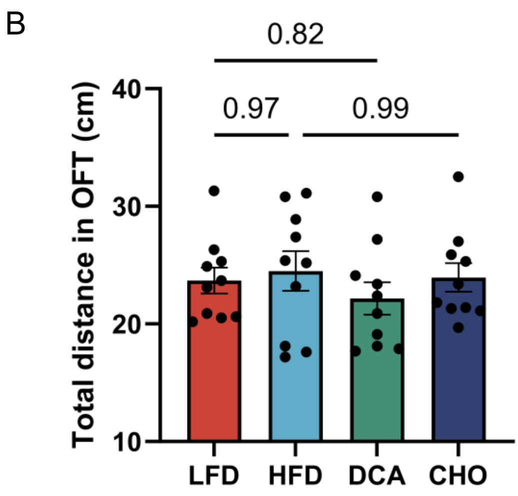

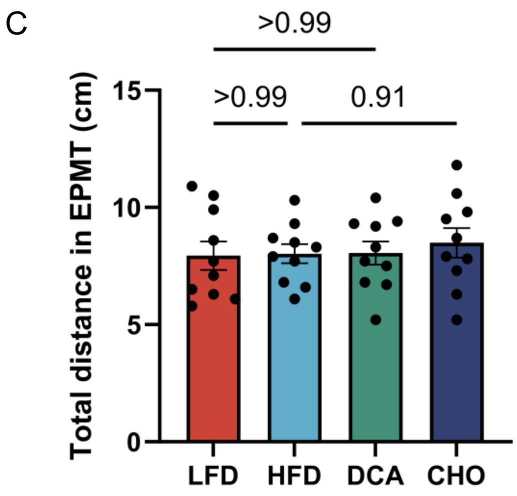

Supplementary Figure 2

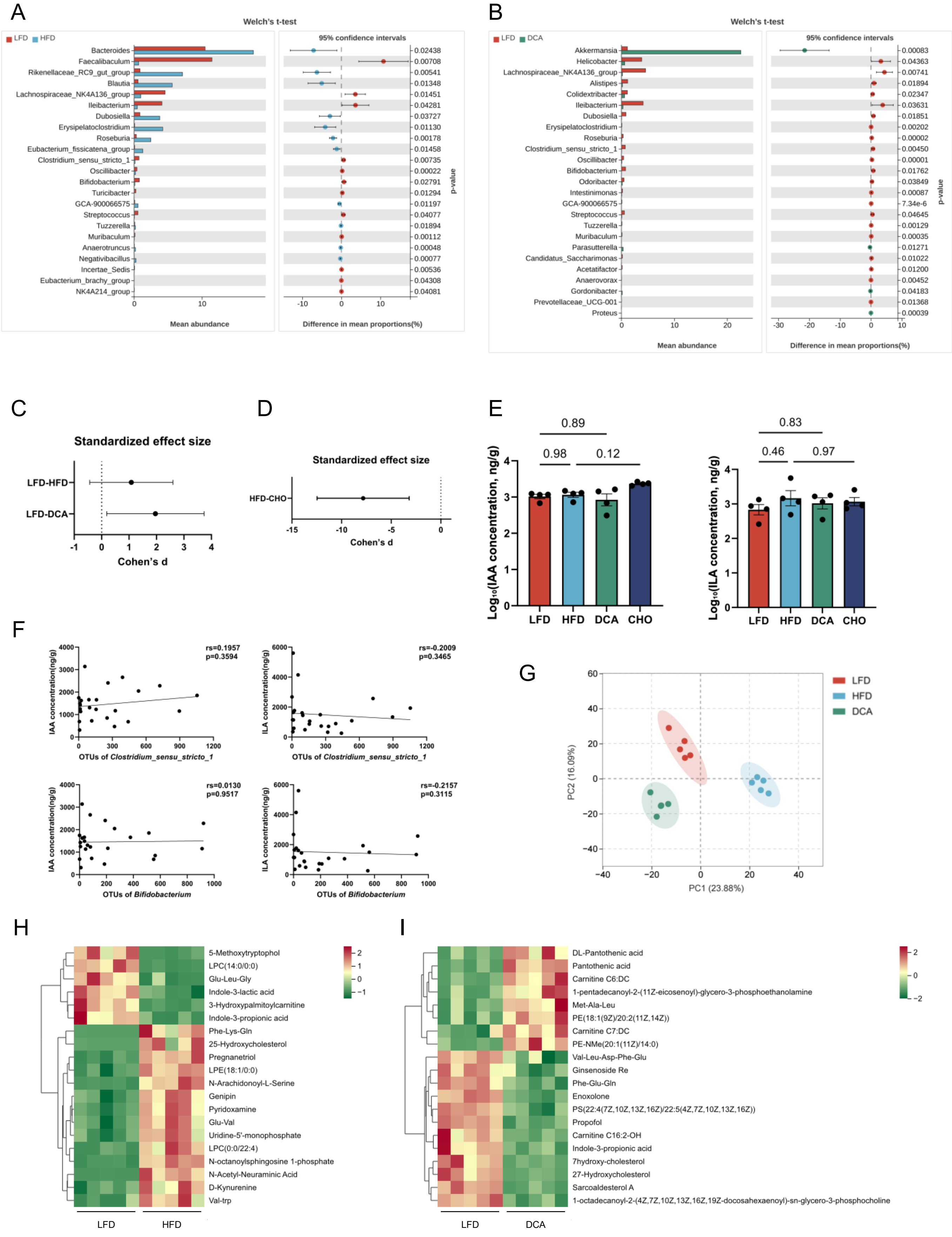

Supplementary Figure 3

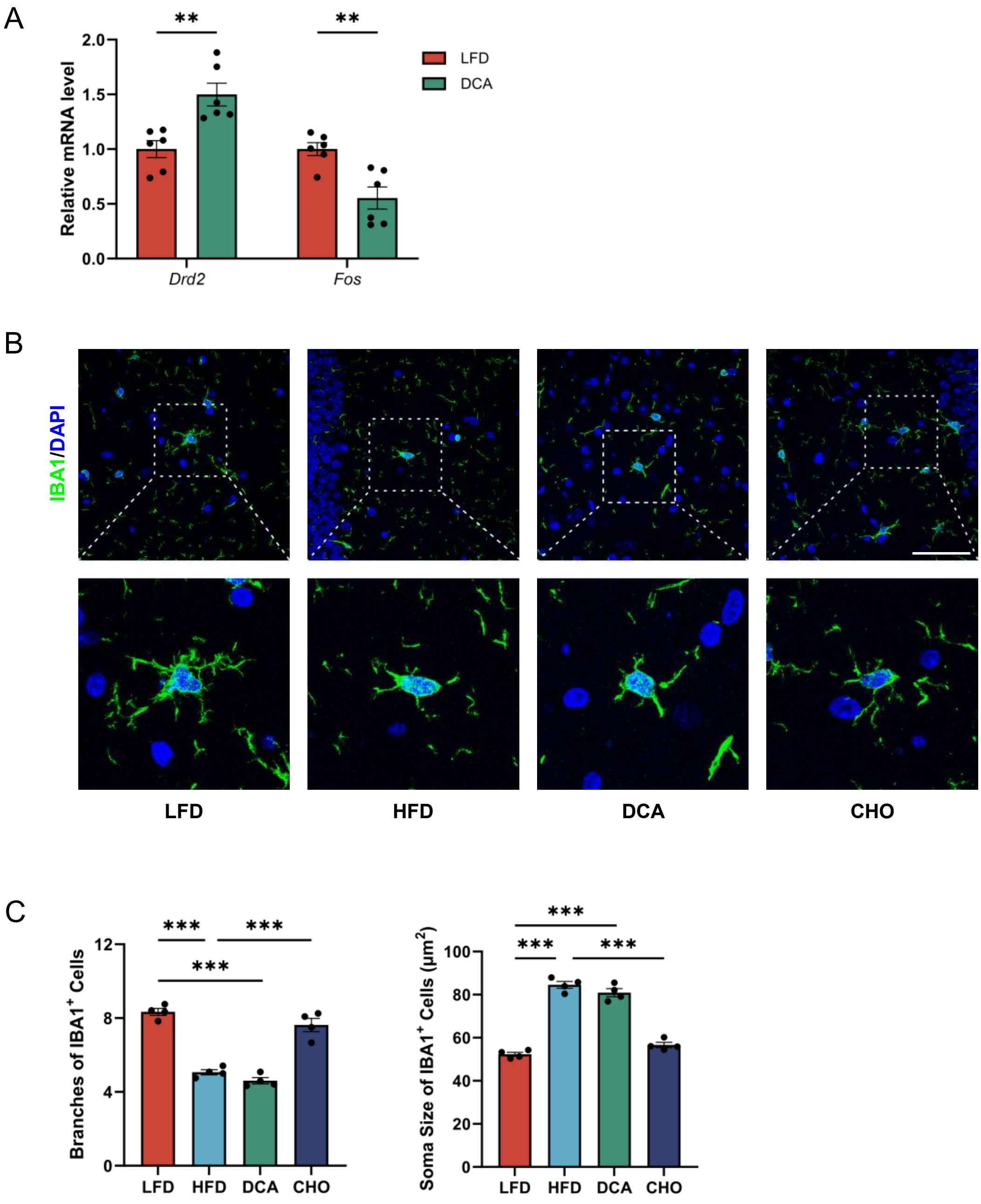

Supplementary Figure 4

A

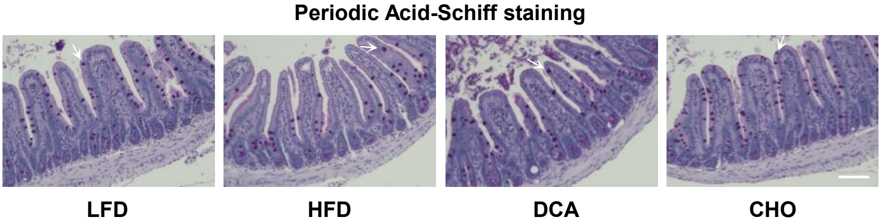

B

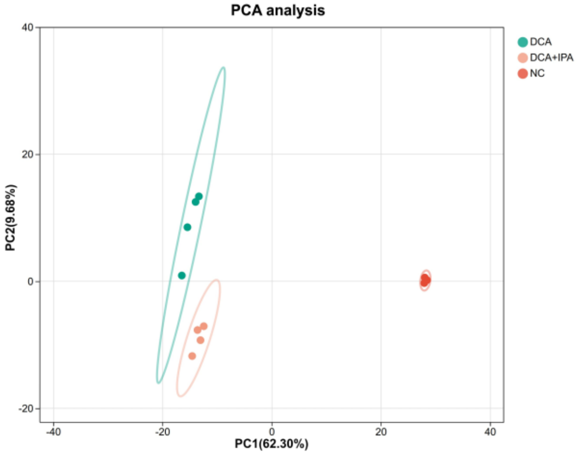

C

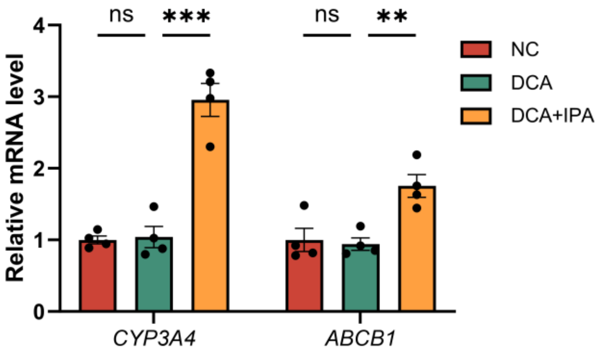

Supplementary Figure 5

A

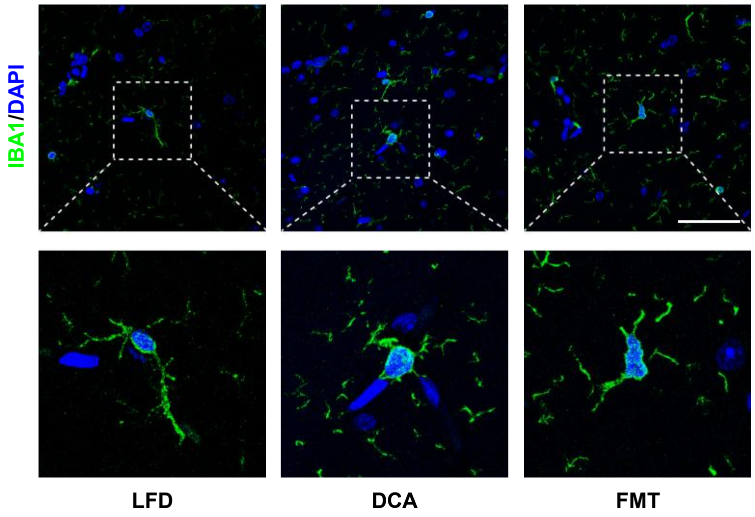

B

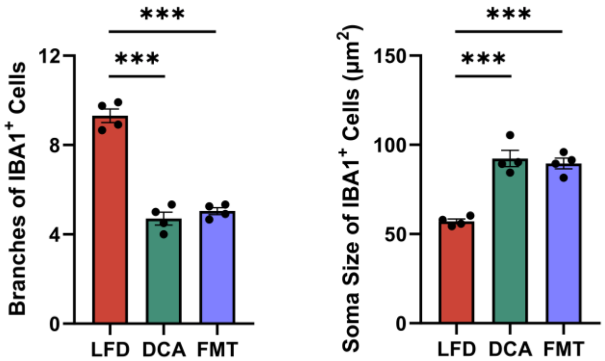

Supplement: Supplementary file 1 [file DataSheet1.pdf]
